# Adaptive Subsets Limit the Anti-Tumoral NK-Cell Activity in Hepatocellular Carcinoma

**DOI:** 10.3390/cells10061369

**Published:** 2021-06-02

**Authors:** Charlotte Rennert, Catrin Tauber, Pia Fehrenbach, Kathrin Heim, Dominik Bettinger, Özlem Sogukpinar, Anita Schuch, Britta Franziska Zecher, Bertram Bengsch, Sven A. Lang, Peter Bronsert, Niklas K. Björkström, Stefan Fichtner-Feigl, Michael Schultheiss, Robert Thimme, Maike Hofmann

**Affiliations:** 1 Department of Medicine II, Faculty of Medicine, University Hospital Freiburg, University of Freiburg, Hugstetter Straße 55, 79106 Freiburg, Germany; charlotte.rennert@uniklinik-freiburg.de (C.R.); Catrin.Tauber@uniklinik-freiburg.de (C.T.); pia.fehrenbach@uniklinik-freiburg.de (P.F.); Kathrin.heim@uniklinik-freiburg.de (K.H.); dominik.bettinger@uniklinik-freiburg.de (D.B.); oezlem.sogukpinar@uniklinik-freiburg.de (Ö.S.); Anita.Schuch@iomedico.com (A.S.); b.zecher@uke.de (B.F.Z.); bertram.bengsch@uniklinik-freiburg.de (B.B.); michael.schultheiss@uniklinik-freiburg.de (M.S.); robert.thimme@uniklinik-freiburg.de (R.T.); 2 Faculty of Biology, University of Freiburg, Schänzlestraße 1, 79104 Freiburg, Germany; 3 Department of Medicine I, Faculty of Medicine, University Medical Centre Hamburg-Eppendorf, University of Hamburg, Martinistraße 52, 20246 Hamburg, Germany; 4 Department of General and Visceral Surgery, Faculty of Medicine, University Hospital Freiburg, University of Freiburg, Hugstetter Straße 55, 79106 Freiburg, Germany; svlang@ukaachen.de (S.A.L.); stefan.fichtner@uniklinik-freiburg.de (S.F.-F.); 5 Department of General, Visceral and Transplantation Surgery, Faculty of Medicine, University Hospital Aachen, University of Aachen, Pauwelsstraße 30, 52074 Aachen, Germany; 6 Institute of Pathology, Faculty of Medicine, University Hospital Freiburg, University of Freiburg, Hugstetter Straße 55, 79106 Freiburg, Germany; peter.bronsert@uniklinik-freiburg.de; 7 Tumorbank, Comprehensive Cancer Center Freiburg, Faculty of Medicine, University of Freiburg, Hugstetter Straße 55, 79106 Freiburg, Germany; 8 Center for Infectious Medicine, Department of Medicine Huddinge, Karolinska Institutet, Karolinska University Hospital, Alfred Nobels Allé 8, 141 52 Huddinge, Sweden; niklas.bjorkstrom@ki.se

**Keywords:** HCC, liver cirrhosis, HCMV, HBV

## Abstract

Hepatocellular carcinoma (HCC) is a global health burden with increasing incidence, poor prognosis and limited therapeutic options. Natural killer (NK) cells exhibit potent anti-tumoral activity and therefore represent potential targets for immunotherapeutic approaches in HCC treatment. However, the anti-tumoral activity of NK cells in HCC associated with different etiologies, and the impact of the heterogeneous NK cell subset, e.g., adaptive and conventional subsets, are not understood in detail. By comparatively analyzing the NK-cell repertoire in 60 HCC patients, 33 liver cirrhosis patients and 36 healthy donors (HD), we show in this study that the NK-cell repertoire is linked to HCC etiology, with increased frequencies of adaptive NK cells in Hepatitis B virus (HBV)-associated HCC. Adaptive NK cells exhibited limited anti-tumoral activity toward liver cancer cells; however, this was not a result of a specific NK-cell impairment in HCC but rather represented an intrinsic feature, since the characteristics of circulating and intra-tumoral adaptive NK cells were conserved between HD, HCC and liver cirrhosis patients. Hence, the expansion of adaptive NK cells with reduced anti-tumoral activity, detectable in HBV-associated HCC, may have implications for tumor surveillance and therapy.

## 1. Introduction

Hepatocellular carcinoma (HCC) is the most common form of primary liver cancer in adults and represents a major health problem due to its increasing incidence worldwide [1,2]. The development of liver cancer is a multifactorial process in which HCC develops from chronic liver damage, resulting in pre-malignant cirrhotic remodeling. Chronic liver damage is frequently caused by alcohol consumption, metabolic syndrome and chronic viral infections, e.g., with hepatitis B virus (HBV) or hepatitis C virus (HCV) [3]. Curative treatment options, including surgical resection or liver transplantation, are limited to early disease stages, while patients suffering from advanced disease can only be treated with transarterial chemoembolization (TACE), systemic therapy, e.g., using sorafenib, or best supportive care [4]. Given these limited therapeutic options and the overall poor prognosis of HCC [1], there is an urgent need for new treatment options. Several studies have indicated a role of innate and adaptive immunity in HCC progression and control [2,3,5], e.g., a longer progression-free survival has been associated with increased frequencies of CD8^+^ T cells targeting tumor-associated antigens [6], and of natural killer cells (NK cells) [7,8]. These data support that both CD8^+^ T cells and NK cells are potent anti-tumoral effector cells, and render these immune cells promising targets for treating HCC. Unlike CD8^+^ T cells, tumor surveillance by NK cells is not mediated by targeting tumor-associated antigens and neo-antigens that are heterogeneously expressed and largely unknown in HCC patients. NK cells mediate anti-tumoral immunity by sensing down-regulated MHC class I expression [9] (“missing-self response” [10]), increased expression of cell stress ligands (“altered-self response” [11,12]), cytokines [13,14,15], and by CD16-mediated antibody-dependent cellular cytotoxicity (ADCC) [16]. While it is therefore known that NK cells represent broadly acting effector cells in tumor surveillance in general [17], anti-tumoral NK-cell activity in HCC associated with different etiologies is not understood in detail.

The human liver is enriched with NK cells that account for up to 40% of all liver immune cells [18,19,20] and that exhibit a considerably different profile compared to circulating NK cells [21,22]. Similarly, high frequencies of NK cells can be found in human liver tumors including HCC [23]. In HCC, phenotypic alterations within the NK-cell repertoire as well as diminished NK-cell effector functions have been reported [24], indicating that failure of the NK-cell response may contribute to tumor growth. Furthermore, distinct phenotypic NK-cell characteristics, e.g., a high expression of cytotoxic granules, have been associated with better survival after treatment [25]. However, a detailed understanding of the NK-cell response in the context of HCC is still missing, especially with respect to heterogeneous NK-cell subsets [26]. The CD56^bright^ NK-cell subset produces cytokines to a high degree, but does not possess cytotoxic properties [27]. In the human liver, the proportion of CD56^bright^ NK cells is significantly higher than in the peripheral blood, including liver-resident subsets [21,28,29]. These liver-resident NK cells exhibit tolerogenic features that partially account for an impaired anti-tumoral NK-cell response in HCC [23,29]. In contrast, CD56^dim^ NK cells represent the predominant circulating NK-cell population [30], which produce cytokines and have a cytotoxic effect [27]. However, the impact of CD56^dim^ NK cells on anti-tumoral activity in HCC is largely unknown.

CD56^dim^ NK cells themselves represent a heterogeneous population, and diversification is best described in association with human cytomegalovirus (HCMV) infection. In association with HCMV, adaptive NK cells emerge that are characterized by a distinct epigenetic, phenotypic and functional profile, including downregulation of the signaling adapter FcɛRIɣ, and a decreased cytokine responsiveness but higher ADCC compared to conventional CD56^dim^ NK cells [31,32,33,34,35]. Previously, our group showed that the emergence of HCMV-associated adaptive NK cells shapes the overall NK-cell response in HBV patients [36]. In this study, we now show that adaptive NK cells are also detectable at higher frequencies in HBV-associated HCC, linking the NK-cell repertoire to HCC etiology. Furthermore, we demonstrate that adaptive NK cells exhibit (i) a limited anti-tumoral activity toward liver cancer cells; (ii) are present in similar frequencies within the blood, tumor tissue and non-tumor liver tissue of HCMV^+^ HCC patients, patients with liver cirrhosis and healthy donors (HD); and (iii) display conserved phenotypic and functional characteristics within the different compartments and among the different cohorts lacking tissue-residency markers. These observations suggest that adaptive NK cells intrinsically harbor reduced anti-tumoral activity against liver cancer cells that is not further impaired in HCC. Taken together, the presence of adaptive CD56^dim^ NK cells may therefore contribute to the impaired anti-tumoral NK-cell activity in HCC, especially in association with HBV infection.

## 2. Materials and Methods

### 2.1. Study Cohort

A cohort of 60 HCC patients (Table 1, Table S1), 36 healthy donors (Table 1, Table S2), 33 patients with liver cirrhosis (Table 1, Table S3) and 28 patients with chronic HBV infection (Table S4) was recruited at the Department of Medicine II of the University Hospital, Freiburg, Germany. Written informed consent was obtained in all cases and the study was conducted according to the Declaration of Helsinki (1975), federal guidelines and local ethics committee regulations (Albert-Ludwigs-University, Freiburg, Germany, approvals 474/14 and 152/17). Peripheral blood mononuclear cells (PBMCs), HCC tumor tissue and adjacent non-tumor liver tissue samples (Table S5) were obtained from the hepatologic and gastroenterologic biobank of the University Hospital, Freiburg (HBUF, banked since 2010) and non-tumoral liver samples (obtained from liver resections due to colon cancer-originated metastasis) from the Karolinska University Hospital, Stockholm (Table S6), and analyzed retrospectively. The donors were not analyzed for genetic polymorphisms in the KLRC2 gene. Due to the restricted availability of patient material, complete analyses including all parameters were not possible for all patients. A detailed list of performed analyses for each donor is included in Tables S1–S6.

### 2.2. PBMC Isolation

PBMCs were isolated from 30–60 mL (depending on availability) EDTA anti-coagulated blood by density-gradient centrifugation, and banked at −80 °C in freezing medium containing 80% fetal calf serum, 10% RPMI culture medium (both Thermo Fisher, Waltham, MA, USA) and 10% DMSO (AppliChem, Darmstadt, HE, Germany) until usage. Frozen PBMCs were then thawed in complete RPMI culture medium (RPMI 1640 supplemented with 10% fetal bovine serum, 1% penicillin/streptomycin and 1.5% 1M HEPES (all Thermo Fisher, Waltham, MA, USA)).

### 2.3. Isolation of Lymphocytes from Tissue Samples

Lymphocytes were isolated from tissue samples by density-gradient centrifugation after mechanical dissociation in complete RPMI culture medium (RPMI 1640 supplemented with 10% fetal bovine serum, 1% penicillin/streptomycin and 1.5% 1M HEPES (all Thermo Fisher, Waltham, MA, USA)).

### 2.4. HCMV Status

HCMV serostatus was determined via HCMV-IgG chemiluminescence immunoassay (DiaSorin LIAISON, Saluggia, VC, Italy) by the Department of Virology, University of Freiburg, for all donors recruited at the University Hospital Freiburg or as previously described by Marquardt et al. [37] for the donors of non-tumoral liver samples from the Karolinska University Hospital, Stockholm, where no plasma/PBMC samples (but mononuclear liver cells) were available. Briefly, 10^6^ mononuclear liver cells were stimulated with 1 µg/mL CMV_pp65_ overlapping peptides (JPT Peptide Technologies, Berlin, BE, Germany) in the presence of Brefeldin A (GolgiPlug (final concentration 5 µL/mL, BD Biosciences, Franklin Lakes, NJ, USA) and Monensin (GolgiStop (final concentration 3.25 µL/mL, BD Biosciences, Franklin Lakes, NJ, USA) overnight (o/n). After 16 h, cytokine production was assessed by flow cytometry [37]. The HCMV seronegative (HCMV) individuals are only included in Figure S1.

### 2.5. Cell Lines

The Huh7 cell line was kindly provided by Volker Lohmann (University Hospital Heidelberg, Germany). The HepG2 cell line was kindly provided by Michael Nassal (University Hospital Freiburg, Germany). The K562 cell line was kindly provided by Hanspeter Pircher (University Hospital Freiburg, Germany). All cell lines were tested for mycoplasma.

### 2.6. Assessment of NK-Cell Function

Degranulation and cytokine production of NK cell subsets were determined after cytokine stimulation o/n with IL-12 (10 ng/mL, Sigma Aldrich, St. Louis, MO, USA) and IL-18 (5 ng/mL, MBL, Woburn, MA, USA), after CD16-crosslinking or after co-culture with the cell lines K562, Huh7 and HepG2 and autologous CD8^+^ T cells (pre-activated with ImmunoCult^TM^ Human CD3/CD28 T Cell Activator (Stemcell Technologies, Vancouver, BC, Canada) according to the manufacturer’s instructions). More detailed information is provided in the supplementary information.

### 2.7. Multiparametric Flow Cytometry

A detailed list of antibodies and reagents used for flow cytometry analysis is included in the supplementary information.

### 2.8. Statistics

Statistical analysis was performed with GraphPad Prism version 9 (GraphPad Software, San Diego, CA, USA). Statistical tests used are indicated in the figure legends. Levels of significance are indicated as follows: *: *p* < 0.05; **: *p* < 0.01; ***: *p* < 0.001; ****: *p* < 0.0001.

## 3. Results

### 3.1. Profiles of Adaptive NK Cells Are Conserved in HCC

CD56^dim^ and CD56^bright^ NK-cell frequencies within the circulating NK-cell population (defined as CD3^−^CD56^+^) were similar in patients with HCC compared to patients with liver cirrhosis and HD (irrespective of HCMV serostatus) with a clear dominance of CD56^dim^ NK cells (Figure S1A–C, Table 1 and Tables S1–S3). A minor but significant reduction of CD56^dim^ NK cells was detectable in cirrhotic patients compared to HD (Figure S1B,D). Frequencies of CD56^dim^ and CD56^bright^ NK-cell populations were not affected by HCMV infection (Figure S1D,E), which was apparent in the majority of our HCC cohort (Figure S1F).

**Figure 1:**
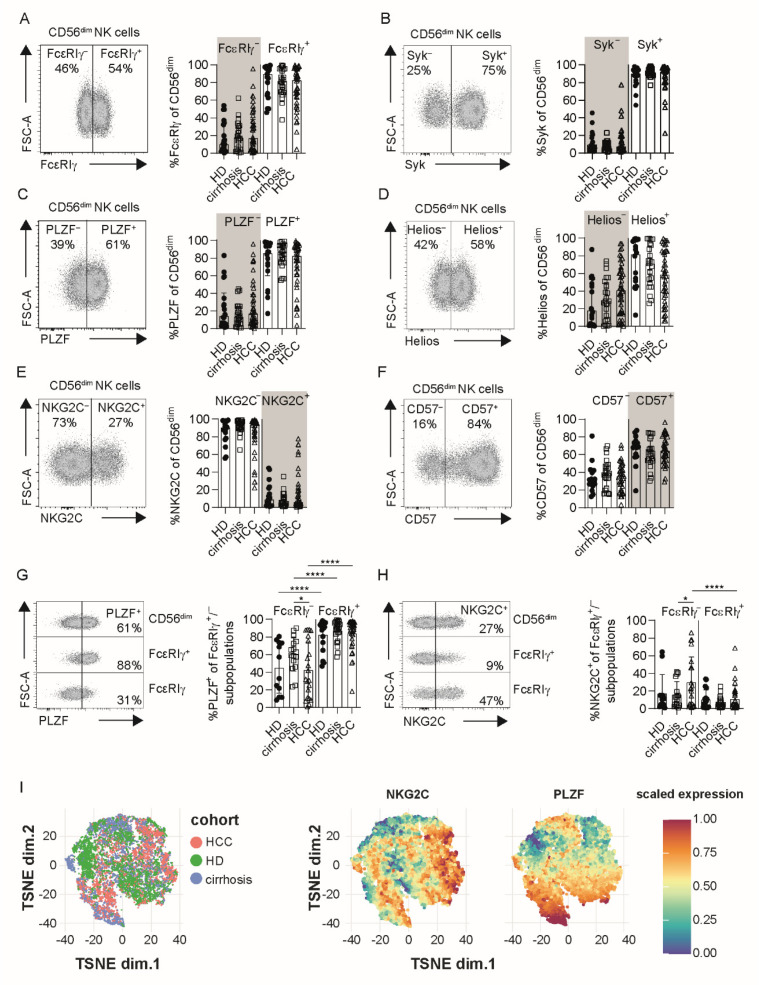
Adaptive NK-cell signatures are conserved in HCC patients. FcεRIγ (**A**), Syk (**B**), PLZF (**C**), Helios (**D**), NKG2C (**E**) and CD57 (**F**) expression on CD56^dim^ NK cells in HCMV^+^ HCC patients (*n* = 42 in (**A**,**D**) *n* = 28 in (**B**), *n* = 40 in (**C**), *n* = 39 in (**E**), *n* = 37 in (**F**)), HD (*n* = 21 in (**A**), *n* = 16 in (**B**,**D**,**F**), *n* = 18 in (**C**,**E**)) and patients with liver cirrhosis (*n* = 24 in (**A**,**C**–**F**), *n* = 18 in (**B**)). PLZF (**G**) and NKG2C (**H**) expression of FcεRIγ^−^ and FcεRIγ^+^ subpopulations in HCC patients (FcεRIγ^−^ *n* = 21, FcεRIγ^+^ *n* = 34), HD (FcεRIγ^−^ *n* = 12, FcεRIγ^+^ *n* = 21) and patients with liver cirrhosis (FcεRIγ^−^ *n* = 16, FcεRIγ^+^ *n* = 24). T-SNE analysis of concatenated flow cytometry data obtained from FcεRIγ^−^ CD56^dim^ NK cells in HCC patients, HD and patients with liver cirrhosis (right). Expression levels of NKG2C and PLZF are plotted on the t-SNE plots (left) (**I**, HCC *n* = 32, cirrhosis *n* = 19, HD *n* = 17). Cells in the grey box correspond to the phenotype of adaptive NK cells (**A**–**F**). Each dot represents an HCMV^+^ individual. Bars indicate median with IQR. Statistical significance for cohort comparison was assessed by using the Kruskal–Wallis test (**A**–**F**) and for subset comparison mixed-effect analysis (**G**,**H**). Patients with less than 10% adaptive FcεRIγ^−^ CD56^dim^ NK cells were excluded (**G**,**H**). HCC: patients with hepatocellular carcinoma, HD: healthy donors, cirrhosis: patients with liver cirrhosis, HCMV: human cytomegalovirus. *: *p* < 0.05; ****: *p* < 0.0001.

Next, we analyzed the presence of FcεRIγ^−^, Syk^−^, PLZF^−^, Helios^−^, NKG2C^+^ and CD57^+^ (Figure 1A–F) cells within CD56^dim^ NK-cell populations in HCMV^+^ HCC patients, to assess the HCMV-associated appearance of adaptive NK cells. HCMV-driven CD56^dim^ NK-cell diversification was also evident in HCMV^+^ HCC patients; however, no significant difference in the frequencies was detectable in comparison to HCMV^+^ patients with liver cirrhosis and HCMV^+^ HD (Figure 1A–F, marked in grey).

Next, we assessed whether the combined phenotypic profile of adaptive NK cells is also conserved in HCMV^+^ HCC patients. For this, the presence of adaptive CD56^dim^ NK cells was defined by more than 10% FcεRIγ^−^ cells among CD56^dim^ NK-cell populations (Figure S1G), and we then comparatively analyzed the expression of PLZF and NKG2C (Figure 1G,H) in FcεRIγ^−^ CD56^dim^ NK cells. Lower frequencies of adaptive FcεRIγ^−^ CD56^dim^ NK cells from HCC patients compared to conventional FcεRIγ^+^ CD56^dim^ NK cells expressed PLZF (Figure 1G), whereas higher frequencies expressed NKG2C (Figure 1H), similar to HCMV^+^ control cohorts. Additional phenotypic analysis of CD2, CD7, Siglec-7, CX3CR1 and CXCR3 expression (Figure S2) also showed a conserved adaptive NK cell profile in HCMV^+^ HCC patients. T-SNE analysis of adaptive FcεRIγ^−^ CD56^dim^ NK cells revealed that the majority of the cells intermingle between all three cohorts (Figure 1I). Thus, HCMV-driven adaptive NK-cell diversification is conserved in HCMV^+^ HCC, compared to HCMV^+^ patients with liver cirrhosis or HCMV^+^ HD.

### 3.2. Adaptive NK Cells in Tumor and Non-Tumor Liver Tissue Exhibit Conserved Profiles

**Figure 2:**
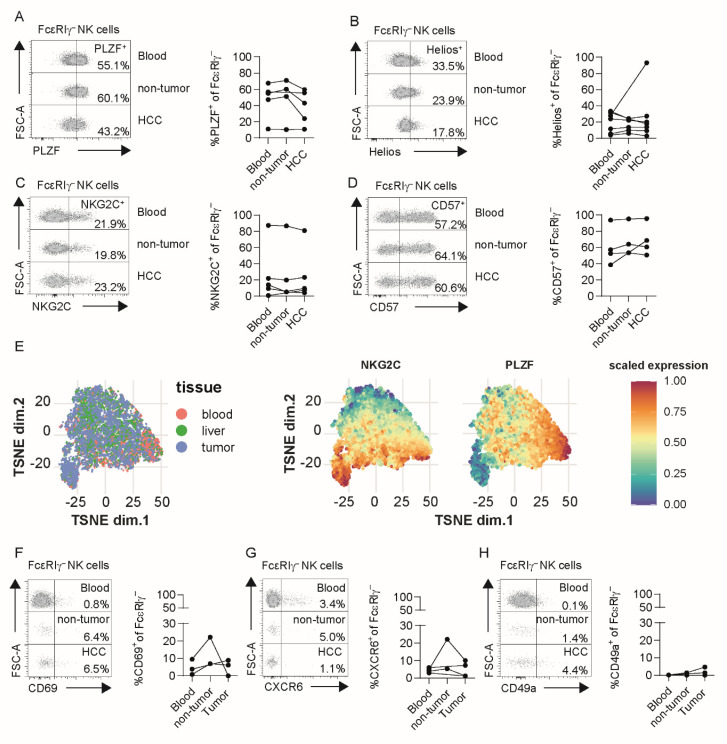
Similar adaptive signature in NK cells isolated from tumor tissue and peripheral blood in HCC patients. PLZF (**A**), Helios (**B**), NKG2C (**C**) and CD57 (**D**) expression of FcεRIγ^−^ CD56^dim^ NK cells in blood (*n* = 5 in **A** and **C**, *n* = 7 in B, *n* = 4 in D), adjacent non-tumoral liver (*n* = 4 for (**A**,**C**), *n* = 6 for (**B**), *n* = 3 for (**D**)) and HCC tissue (*n* equal to blood) of HCMV^+^ HCC patients. T-SNE analysis of concatenated flow cytometry data obtained from FcεRIγ^−^ CD56^dim^ NK cells in blood (*n* = 5), adjacent non-tumoral liver (*n* = 4) and HCC tissue (*n* = 5) of HCMV^+^ HCC patients (**E**). Expression levels of NKG2C and PLZF are depicted in the t-SNE plots (left). CD69 (**F**), CXCR6 (**G**) and CD49a (**H**) expression of FcεRIγ^−^ CD56^dim^ NK cells in blood (*n* = 3 for (**F**,**H**), *n* = 4 for (**G**)), non-tumor (*n* = 2) and HCC tissue (*n* equal to blood) of HCMV^+^ HCC patients. Each point represents a single HCMV^+^ patient, and lines connect the samples from one patient. Bars indicate median with IQR. Statistical significance was tested using the Kruskal–Wallis test (**A**–**D**) and mixed-effect analysis (**F**–**H**). HCC: hepatocellular carcinoma.

As the human liver is enriched with NK cells, we analyzed non-tumor liver tissue (Tables S5 and S6) and HCC tumor samples (Table S5) for the presence of adaptive FcεRIγ^−^ CD56^dim^ NK cells in HCMV^+^ patients. For this, we stained for FcεRIγ (Figure S3A), PLZF (Figure S3B), Helios (Figure S3C), NKG2C (Figure S3D) and CD57 (Figure S3E) in CD56^dim^ NK cells obtained from liver tissues. Indeed, we could detect CD56^dim^ NK cells lacking FcεRIγ (Figure S3A), PLZF (Figure S3B) or Helios (Figure S3C), and expressing NKG2C (Figure S3D) or CD57 (Figure S3E) in non-tumoral liver tissue and HCC tumor tissue. To assess whether adaptive NK cells isolated from the tumor are phenotypically different, we compared the expression of PLZF, Helios, NKG2C and CD57 in FcεRIγ^−^ CD56^dim^ NK cells from matched blood, adjacent non-tumor liver tissues, and HCC tumor tissues obtained from HCMV^+^ HCC patients with more than 10% FcεRIγ^−^ adaptive CD56^dim^ NK cells in their blood. All analyzed markers were equally expressed in FcεRIγ^−^ adaptive CD56^dim^ NK cells from the three matched compartments of the respective donors, suggesting conserved profiles even within HCC tumors (Figure 2A–D). This finding was further corroborated by t-SNE analysis of FcεRIγ^−^ adaptive CD56^dim^ NK, showing no clear separation between the cells of the different compartments (Figure 2E). To further address whether FcεRIγ^−^ CD56^dim^ NK cells are circulating or liver-infiltrating cells, or rather, represent tissue-resident NK cells, we analyzed the expression of the tissue-resident marker molecules CD69, CXCR6 and CD49a on CD56^dim^ NK-cell subpopulations and CD56^bright^ NK cells. FcεRIγ^−^ adaptive CD56^dim^ NK cells, isolated from liver tissue irrespective of non-tumor or tumor origin, displayed a low expression of these tissue-resident markers (Figure 2F–H), whereas CD56^bright^ NK cells obtained from liver tissue expressed higher levels of CD69, CXCR6 and CD49a (Figure S4). These findings were confirmed by cross-sectionally analyzing blood, non-tumor liver and HCC tumor tissues (Figure S5). Hence, adaptive CD56^dim^ NK cells found in HCC lesions are most probably circulating or tumor-infiltrating lymphocytes.

### 3.3. Limited Anti-Tumoral Activity of FcεRIγ^−^ Adaptive NK Cells

**Figure 3:**
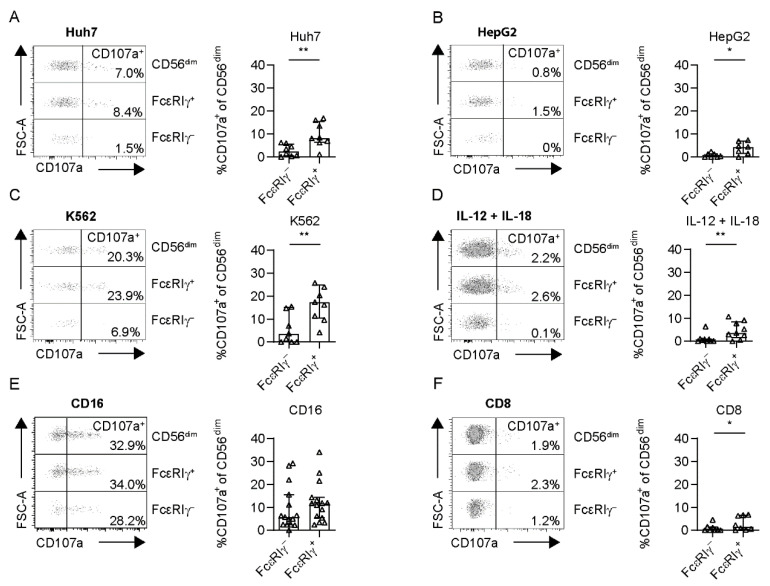
Functional capacity of adaptive versus conventional NK cells in HCC patients. CD107a expression of CD56^dim^ NK cells following stimulation with Huh7 (**A**) (*n* = 8), HepG2 (**B**) (*n* = 7) or K562 (**C**), *n* = 9) cell lines for 5 h, cytokine stimulation with IL-12 and IL-18 overnight (**D**) (*n* = 9) CD16 crosslink (**E**) (*n* = 8), or stimulation with activated autologous CD8^+^ T cells for 5 h (**F**) (*n* = 8) in HCMV^+^ HCC patients. Each dot represents an HCMV^+^ individual. Bars indicate median with IQR. Patients with less than 10% adaptive FcεRIγ^−^ CD56^dim^ NK cells were excluded. The following statistical analyses were performed: two-tailed Wilcoxon Test. *: *p* < 0.05; **: *p* < 0.01.

To assess the anti-tumoral capacity, we performed co-culture experiments of circulating FcεRIγ–based NK-cell populations of HCMV^+^ HCC patients (>10% FcεRIγ^−^ adaptive CD56^dim^ NK cells) with the hepatoma cell lines Huh7 and HepG2. CD107a expression, as a surrogate marker for degranulation (Figure 3A,B), and MIP-1ß (Figure S6A,B) production after stimulation with Huh7 or HepG2 cells, were significantly reduced in FcεRIγ^−^ adaptive NK cells compared to conventional FcεRIγ^+^ NK cells, with the same trends seen for IFN-γ (Figure S7A,B). This observation indicates reduced anti-tumoral activity against liver cancer cells. We also observed the same effect after stimulation with the leukemia cell line K562 (Figure 3C, Figures S6C and S7C).

In addition, FcεRIγ^−^ adaptive NK cells exhibited reduced IL-12 + IL-18 responsiveness mirrored by cytokine secretion (Figures S6D and S7D) and also by diminished degranulation (Figure 3D).

However, CD16-mediated functionality, tested by CD107a and cytokine expression after cross-linking, was comparable between adaptive FcεRIγ^−^ and conventional FcεRIγ^+^ CD56^dim^ NK cells in HCMV^+^ HCC patients (Figure 3E, Figures S6E and S7E). Finally, to analyze the immunoregulatory potential of FcεRIγ^−^ adaptive NK cells compared to FcεRIγ^+^ conventional NK cells toward CD8^+^ T cells, we co-cultivated FcεRIγ–based NK-cell populations from HCC patients with autologous, activated CD8^+^ T cells. Degranulation (Figure 3F) by FcεRIγ^−^ CD56^dim^ NK cells was significantly reduced compared to FcεRIγ^+^ CD56^dim^ NK cells, with a similar trend with respect to cytokine production (Figures S6F and S7F), suggesting a reduced immunoregulatory activity of adaptive NK cells toward CD8^+^ T cells. Representative negative controls for all assays are shown in Figure S8. In summary, while reduced immunomodulatory function may potentially lead to indirect support of the CD8^+^ T cell-based anti-tumoral response in HCC, the direct anti-tumoral activity mediated by adaptive NK cells is limited.

### 3.4. Adaptive NK Cells Are Not Specifically Impaired in HCC

**Figure 4:**
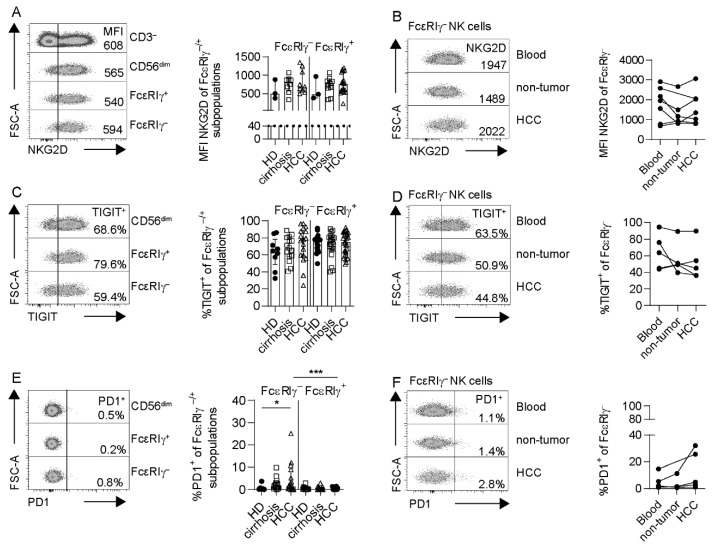
Checkpoint receptor expression of NK cells in HCC patients. MFI of NKG2D (**A**,**B**), expression of TIGIT (**C**,**D**) and PD1 after deduction of FMO (**E**,**F**) of FcεRIγ-subpopulations in HCC patients (FcεRIγ^−^ *n* = 9 in (**A**), *n* = 18 in (**C**), *n* = 16 in (**E**), FcεRIγ^+^ *n* = 17 in (**A**), *n* = 30 in (**C**), *n* = 25 in (**E**)), HD (FcεRIγ^−^ *n* = 3 in (**A**), *n* = 9 in (**C**), *n* = 6 in (**E**), FcεRIγ^+^ *n* = 3 in (**A**), *n* = 18 in (**C**), *n* = 13 in (**E**)) and patients with liver cirrhosis (FcεRIγ^−^ *n* = 8 in (**A**), *n* = 15 in (**C**), *n* = 14 in (**E**), FcεRIγ^+^ *n* = 11 in (**A**), *n* = 22 in (**C**), *n* = 18 in (**E**)) (left) and in HCC patients’ blood (*n* = 7 in (**B**), *n* = 5 in (**D**,**F**)) compared to non-tumor (*n* = 6 in (**B**), *n* = 4 in (**D, F**)) and HCC lesion (*n* equal to blood) (right). Each dot represents an HCMV^+^ individual with more than 10% adaptive FcεRIγ^−^ CD56^dim^ NK cells, and the line connects the samples from one patient. Bars indicate median with IQR. Statistical significance was tested by using mixed-effect analysis (**A**,**C**,**E**) and the Kruskal–Wallis test (**B**,**D**,**F**). HCC: patients with hepatocellular carcinoma, HD: healthy donors, cirrhosis: patients with liver cirrhosis. *: *p* < 0.05; ***: *p* < 0.001.

Next, we investigated whether the HCC-associated adaptive NK cells exhibit characteristics of specific impairment. First, we analyzed the expression of NKG2D on FcεRIγ–based CD56^dim^ NK-cell populations, relevant in tumor-associated NK activation. We did not detect differences in NKG2D expression, neither on FcεRIγ^−^ adaptive nor on FcεRIγ^+^ conventional NK cells from HCC patients, compared to control groups (Figure 4A). NKG2D expression on adaptive NK cells did not differ between matched samples obtained from blood, adjacent non-tumor liver and HCC tumor tissue (Figure 4B). NKp46 and NKp30 expressions were also comparable on FcεRIγ^−^ adaptive NK cells obtained from HCC patients and the control groups (Figure S9A,B).

**Figure 5:**
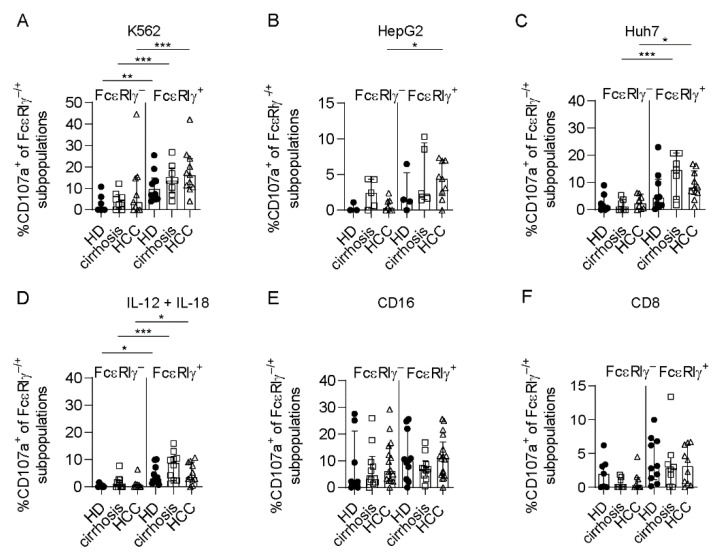
Conserved functionality of CD56^dim^ NK-cell subpopulations. CD107a expression of FcεRIγ-subpopulations in HCC patients, HD and patients with liver cirrhosis after stimulation with K562 (**A**), (HCC FcεRIγ^−^ *n* = 9, HCC FcεRIγ^+^ *n* = 11, HD FcεRIγ^−^ *n* = 7, HD FcεRIγ^+^ *n* = 10, cirrhosis FcεRIγ^−^ and FcεRIγ^+^ *n* = 8), HepG2 (**B**), (HCC FcεRIγ^−^ *n* = 7, HCC FcεRIγ^+^ *n* = 9, HD FcεRIγ^−^ *n* = 4, HD FcεRIγ^+^ *n* = 3, cirrhosis FcεRIγ^−^ and FcεRIγ^+^ *n* = 5) and Huh7 (**C**), (HCC FcεRIγ^−^ *n* = 8, HCC FcεRIγ^+^ *n* = 10, HD FcεRIγ^−^ *n* = 7, HD FcεRIγ^+^ *n* = 9, cirrhosis FcεRIγ^−^ and FcεRIγ^+^ *n* = 7) cell lines, cytokines (**D**), (HCC FcεRIγ^−^ *n* = 9, HCC FcεRIγ^+^ *n* = 11, HD FcεRIγ^−^ *n* = 8, HD FcεRIγ^+^ *n* = 11, cirrhosis FcεRIγ^−^ and FcεRIγ^+^ *n* = 10), CD16 crosslink (**E**), (HCC FcεRIγ^−^ *n* = 15, HCC FcεRIγ^+^ *n* = 17, HD FcεRIγ^−^ *n* = 8, HD FcεRIγ^+^ *n* = 11, cirrhosis FcεRIγ^−^ and FcεRIγ^+^ *n* = 10) or autologous CD8^+^ T cells (**F**), (HCC FcεRIγ^−^ *n* = 8, HCC FcεRIγ^+^ *n* = 10, HD FcεRIγ^−^ *n* = 7, HD FcεRIγ^+^ *n* = 10, cirrhosis FcεRIγ^−^ and FcεRIγ^+^ *n* = 8). Each dot represents an HCMV^+^ individual with more than 10% adaptive FcεRIγ^−^ CD56^dim^ NK cells. Bars indicate median with IQR. Statistical significance was tested by using mixed-effect analysis. HCC: patients with hepatocellular carcinoma, HD: healthy donors, cirrhosis: patients with liver cirrhosis. *: *p* < 0.05; **: *p* < 0.01; ***: *p* < 0.001.

We also tested for checkpoint molecule expression. While TIGIT expression on FcεRIγ^−^ CD56^dim^ NK cells was comparable to FcεRIγ^+^ CD56^dim^ NK cells (Figure 4C), a higher fraction of FcεRIγ^−^ adaptive CD56^dim^ NK cells expressed PD1 (Figure 4E). PD1 expression was significantly increased on FcεRIγ^−^ adaptive CD56^dim^ NK cells from HCMV^+^ HCC patients, compared to HCMV^+^ HD. Expression of both TIGIT and PD1 did not differ between matched blood, adjacent non-tumor liver tissue and tumor tissue (Figure 4D,F). Additionally, no significant differences in NKG2A expression were detected between CD56^dim^ NK cell subpopulations in HCMV^+^ HCC and control groups (Figure S9C). By comparing the functional capacities, we did not observe differences in the degranulation (Figure 5) and cytokine secretion (Figure S10) of FcεRIγ^−^ adaptive NK cells obtained from HCMV^+^ HCC patients compared to HCMV^+^ control cohorts, neither after stimulation with tumor cells (Figure 5A–C, Figure S10A–F), nor with cytokines (Figure 5D, Figure S10G,H), nor after CD16 crosslink (Figure 5E, Figure S10K,L), nor after co-cultivation with activated autologous CD8^+^ T cells (Figure 5F, Figure S10I,J). Thus, we did not observe functional impairment of adaptive and conventional CD56^dim^ NK cells in HCMV^+^ HCC.

### 3.5. Adaptive NK Cells Are Expanded in HCMV^+^ HBV-Associated HCC Patients

**Figure 6:**
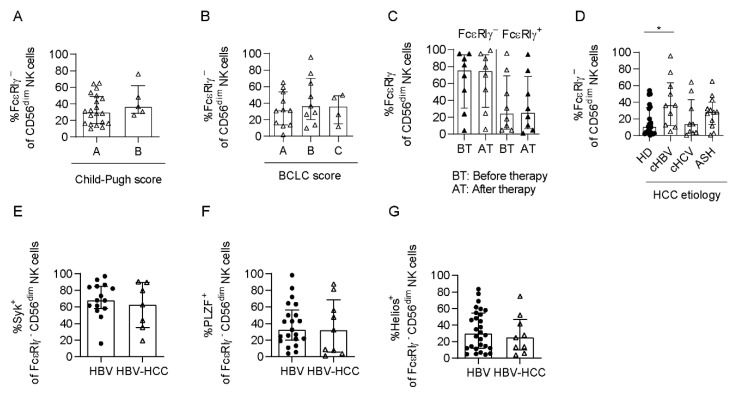
Expansion of FcεRIγ^−^ adaptive NK cells in HBV-associated HCMV+ HCC patients. Frequency of FcεRIγ^−^ adaptive NK cells in HCC patients depending on Child scores (**A**) (*n* = 20 for Child A, *n* = 5 for Child B), BCLC scores (**B**) (*n* = 11 for BCLC A, *n* = 9 for BCLC B, *n* = 4 for BCLC C) HCC therapy (**C**) (*n* = 8), and underlying HCC etiology (**D**) (HD *n* = 21, cHBV HCC *n* = 10, cHCV HCC *n* = 9, ASH HCC *n* = 12). Syk (**E**), PLZF (**F**) and Helios (**G**) expression on FcεRIγ^−^ adaptive CD56^dim^ NK cells in HCMV^+^ HBV infected patients (*n* = 15 in (**E**), *n* = 21 in (**F**), *n* = 28 in (**G**)) and HCMV^+^ HBV-associated HCC patients (*n* = 7 in (**E**), *n* = 9 in (**F**,**G**)). Each dot represents an HCMV^+^ individual with more than 10% adaptive FcεRIγ^−^ CD56^dim^ NK cells. Bars indicate median with IQR. The following statistical analyses were performed: unpaired, two-tailed Mann–Whitney test (**A**,**E**–**G**), Kruskal–Wallis test (**B**,**D**), paired two-tailed Wilcoxon Test (**C**). ASH: alcohol-induced steatohepatitis, AT: after therapy, BT: before therapy, HBV: hepatitis B virus, HBV-HCC: HBV-associated HCC patients, HCC: patients with hepatocellular carcinoma, HCMV: human cytomegalovirus, HCV: hepatitis C virus, HD: healthy donors. *: *p* < 0.05.

Lastly, we assessed whether the frequencies of FcεRIγ^−^ CD56^dim^ NK cells are associated with clinical parameters. We did not detect different frequencies of FcεRIγ^−^ CD56^dim^ NK cells dependent on the Child–Pugh score, reflecting the severity of cirrhosis and liver function (Figure 6A), and the BCLC score (Figure 6B) used to stage HCC. Similarly, no significant correlation of FcεRIγ^−^ CD56^dim^ NK-cell frequencies with the tumor marker AFP could be observed (Figure S11, Table S1). We also did not observe changes in the frequencies of FcεRIγ^+^ or FcεRIγ^−^ CD56^dim^ NK cells induced by ablative therapy (Figure 6C).

Next, we investigated the impact of the underlying HCC etiology on the presence of adaptive NK cells in HCMV^+^ HCC patients. Adaptive NK-cell frequencies were significantly increased in HBV-associated HCMV^+^ HCC patients compared to HCMV^+^ HD but not in HCMV^+^ HCV- or ASH-associated HCC (Figure 6D). Thus, HBV infection also alters the NK-cell repertoire in HCMV^+^ HCC patients. Notably, Syk (Figure 6E), PLZF (Figure 6F) and Helios (Figure 6G) expression, cytokine stimulation (Figure S12A), and ADCC (Figure S12B) were similar in FcεRIγ^−^ adaptive NK cells from patients with chronic HBV infection (non-cirrhotic) and with HBV-associated HCC (Tables S1 and S4). Hence, the etiology is a relevant determinant of the NK-cell repertoire in HCC patients.

## 4. Discussion

Our comparative analysis of the NK cell repertoire in HCC and liver cirrhosis patients and in HD revealed that the presence of adaptive CD56^dim^ NK cells, e.g., defined by FcεRIγ^−^, in HCC and pre-malignant liver cirrhosis was clearly associated with HCMV infection, as previously reported in other patient cohorts including cohorts affected by viral infections or leukemia [22,31,32,33,34,36,38,39,40]. Since HCMV infection is frequent [41,42], as also reflected by our cohorts of patients suffering from HCC and cirrhosis, the HCMV-associated emergence of adaptive CD56^dim^ NK cells is an important parameter to consider in NK cell-mediated cancer immunosurveillance with potential clinical impact. However, a sound correlation analysis between HCMV serostatus and patient outcome was not possible in our study, due to the cohort size.

Besides this link to HCMV, we also detected increased frequencies of adaptive CD56^dim^ NK cells in HBV-associated HCC compared to the other analyzed etiologies, such as HCV and ASH. Furthermore, no other tested clinical parameters, including liver function or tumor stage, correlated with the frequencies of adaptive CD56^dim^ NK cells in our cohort, arguing against a general HCMV reactivation in HCC lesions accompanied by an expansion of adaptive CD56^dim^ NK cells. The underlying mechanisms of the pronounced expansion of adaptive CD56^dim^ NK cells seem rather to be HBV-related. This is in line with a recent report from our group, showing increased frequencies of FcεRIγ^−^ adaptive CD56^dim^ NK cells in patients chronically infected with HBV, compared to HCV and HD [36]. However, further studies are required to uncover these HBV-related mechanisms. Possible mechanisms may include the presence of anti-HCMV/anti-HBV antibodies, and selection for specific HCMV strains, cytokines and also genetic factors [36]. Interestingly, Wijaya et al. also demonstrated expansion of KLRG1^+^ NK cells in the context of chronic HCV and HBV infection without an association with HCMV infection [43,44,45]. However, the lack of FcεRIγ^−^ adaptive NK cells in nearly all HCMV negative patients in our cohort argues against the induction of this adaptive NK cell subset by HBV alone.

FcεRIγ^−^ adaptive CD56^dim^ NK cells are themselves also a heterogeneous population, as exemplified by previously described FcεRIγ^−^/NKG2C or FcεRIγ^−^/Helios co-expression analyses [36,46]. Our results show that the phenotypic and functional characteristics of FcεRIγ^−^ adaptive CD56^dim^ NK cells are largely conserved in HCC and control cohorts. Clearly, further high-dimensional analyses, e.g., single-cell RNA sequencing, are required to fully elucidate the entire heterogeneity of HCC-associated adaptive CD56^dim^ NK cells, especially with respect to different etiologies. A high degree of conserved characteristics and similar frequencies of adaptive NK cells were also detectable within tumor and non-tumor liver tissue and blood. These observations indicate that adaptive CD56^dim^ NK cells whose presence in the liver has also previously been described [46], are, rather, circulating lymphocytes instead of tissue-resident subsets. This assumption is also in line with our findings that adaptive CD56^dim^ NK cells exhibited only a low expression of tissue-resident markers [20,28,29,37] like CXCR6, CD69 and CD49a. Hence, adaptive CD56^dim^ NK cells found within HCC tissue likely represent circulating/tumor-infiltrating lymphocytes that can be monitored and targeted via the blood. This observation may have important relevance for translational applications, such as for immunotherapeutic interventions. However, additional studies are required to further clarify the circulating/infiltrating nature of adaptive CD56^dim^ NK cells within the liver, e.g., by the analysis of the KIR repertoire, to exclude that adaptive NK cells are locally expanded.

Compared to the conventional FcεRIγ^+^ CD56^dim^ NK-cell subset, adaptive FcεRIγ^−^ CD56^dim^ NK cells displayed reduced direct anti-tumoral activity. This limited anti-tumoral activity was not only evident against the leukemia cell lines K562 or Raji, as reported previously in naturally occurring adaptive NK cells and FcεRIγ^−^ NK cells produced in vitro by gene editing [35,46,47], but also as unraveled by this study against liver cancer cells reflecting solid tumors. This observation may indicate an inherently reduced adaptive NK cell response triggered, e.g., by low MHCI expression. This limited response of adaptive CD56^dim^ NK cells toward MHCI^low^ target cells may be detrimental in cancer immunosurveillance, reflecting a possible successful cancer evasion strategy. However, it also potentially represents a mechanism of tissue protection, especially in the liver, since hepatocytes express low levels of MHCI [48]. In line with this, Oh et al. reported that the presence of adaptive NK cells was associated with less severe liver damage [49]. A general underlying mechanism is further supported by the observation that we could not detect differences in the anti-tumoral activity of patients with different etiologies of HCC and that adaptive CD56^dim^ NK cells from HCC patients and HD exhibited a similarly limited anti-tumoral activity. Thus, it is probably not a specific impairment in HCC, but rather an intrinsic and stable characteristic of adaptive CD56^dim^ NK cells. This also fits our data regarding an unaltered expression of NKG2D, TIGIT and NKG2A on adaptive CD56^dim^ NK cells obtained from HCC patients compared to HD, which have all been reported to be relevant in mediating the anti-tumoral NK cell response [11,12,50,51]. In contrast to adaptive CD56^dim^ NK cells, the HCC-associated dysfunction observed within the liver-resident CD56^bright^ NK-cell population is at least in part directly induced by HCC in a contact-dependent manner [23].

Expression of PD1, a checkpoint inhibitor molecule, was also described on CD56^dim^ NK cells of cancer patients and healthy controls [52,53]. In particular, Liu et al. reported elevated PD1 expression on NK cells obtained from HCC patients [53]. Furthermore, in another study analyzing HCMV seropositive individuals, PD1^+^ NK cells exhibited a higher expression of CD57 [52], a maturation marker that is also highly expressed on adaptive CD56^dim^ NK cells [31]. In line with these reports, we detected an increased expression of the checkpoint inhibitor PD1 on adaptive cells compared to conventional CD56^dim^ NK cells that was significantly higher in HCC patients compared to HD. However, the overall frequency of PD1 expressing CD56^dim^ NK cells was extremely low in the blood, adjacent non-tumor, and tumor liver tissue of HCC patients. Of note, tumor-infiltrating NK cells in mice and colon cancer patients also showed low expression rates of PD1 [50], questioning a general direct role of PD1 as a checkpoint inhibitor of NK cells. Hence, we assume a minor role for this checkpoint inhibitor molecule in adaptive CD56^dim^ NK cell-mediated immunosurveillance in HCC [54].

In addition, as in liver cirrhosis patients and HD, adaptive CD56^dim^ NK cells obtained from HCC patients displayed reduced cytokine responsiveness, as described previously in other settings [31,32,33,34,35,36], and decreased immunoregulatory function toward activated CD8^+^ T cells. The decreased immunoregulatory function of adaptive CD56^dim^ NK cells may result from the low NCR expression. This is in line with previous reports in the LCMV mouse system [55,56,57], showing, for example, that an altered regulation of CD8^+^ T cells by NK cells in FcεRIγ^-/-^ mice resulted in better survival. However, it remains unclear whether the FcεRIγ^-/-^ NK cells in this mouse model are comparable to their human counterparts since, in the murine system, adaptive/memory-like NK cells are defined by other features such as Ly49H expression. In summary, in the context of cancer immunosurveillance, the conserved functional features of FcεRIγ^−^ adaptive NK cells may favor tumor-specific immunity by T cells at the expense of a broad, less specific NK cell-mediated anti-tumoral defense induced by altered-self, missing-self and bystander/cytokine-mediated mechanisms, also in HCC. The observation that adaptive CD56^dim^ NK cells are more frequent in HBV-associated HCC therefore suggests that HCC etiology is linked to differences in cancer immunosurveillance, a fact that needs to be considered in the design and application of immunotherapies in HCC.

## Figures and Tables

**Table 1:** Characteristics of the study cohort.

Cohort	Age in Years (Median/Range)	Sex (m/f)	Etiology (HBV/HCV/ASH/NASH/Others ^1^)	Child Score (A/B/C/No Cirrhosis/n.d.)	BCLC Score (0/A/B/C/n.d.)	HCMV Serostatus (pos/neg/n.d.)
HCC	67/45–84	50/10	13/13/19/8/7	39/10/3/7/1	1/23/26/8/2	42/18/0
cirrhosis	61/45–78	20/13	3/6/12/8/4	14/11/8/0/0	n.a.	24/8/1
HD	58/26–86	15/21	n.a.	n.a.	n.a.	22/14/0

^1^ Other etiologies: autoimmune hepatitis, hemochromatosis, drug toxic, secondary biliary cirrhosis, alpha 1-antitrypsin deficiency, steatosis hepatitis, n.d. Abbreviations: HCC: patients with hepatocellular carcinoma, HD: healthy donor, HBV: hepatitis B virus, HCV: hepatitis C virus, ASH: alcoholic steatohepatitis, NASH: non-alcoholic steatohepatitis, BCLC: Barcelona Clinic Liver Cancer, HCMV: human cytomegalovirus, n.a.: not applicable, n.d.: not determined.

## Data Availability

The data presented in this study are available on request from the corresponding author.

## Supplementary Materials

The supplementary information includes supplementary methods and figures, as well as detailed study cohort characteristics. The following are available online at https://www.mdpi.com/article/10.3390/cells10061369/s1, Supplemental Figure S1. Increased frequency of FcεRIγ- adaptive NK cells in HCMV+ HCC patients. Figure S2. Adaptive NK-cell profile is comparable between HCC patients and control cohorts. Figure S3. Adaptive NK-cell repertoire in blood and tissue of HCC patients. Figure S4. CD56^dim^ NK cells do not express tissue residency markers in matched tumor/non-tumor HCC tissue. Figure S5. CD56^dim^ NK cells do not express tissue residency markers in liver tissue. Figure S6. MIP-1ß production by FcεRIγ- adaptive and FcεRIγ+ conventional NK cells in HCC patients. Figure S7. IFNγ production by FcεRIγ- adaptive and FcεRIγ+ conventional NK cells in HCC patients. Figure S8. Negative controls for functional NK-cell studies. Figure S9. NCR and NKG2A expression of adaptive NK cells in HCC patients. Figure S10. Conserved cytokine production of CD56dim NK-cell subpopulations. Figure S11. Frequency of FcεRIγ- adaptive NK cells do not correlate with AFP values. Figure S12. Conserved functional profile of FcεRIγ- adaptive CD56dim NK cells between HCMV+ HBV and HCMV+ HBV-associated HCC patients. Table S1. Study cohort of HCC patients (PBMCs). Table S2. Study cohort of healthy donors (HD). Table S3. Study cohort of patients with liver cirrhosis. Table S4. Study cohort of patients with chronic HBV infection (HBV). Table S5. Study cohort of liver and tumor samples from the hepatologic and gastroenterologic biobank of the University hospital Freiburg. Table S6. Study cohort of liver samples obtained from the Karolinska Institute, Sweden.
