# Supplementary material for: Adaptive Subsets Limit the Anti-Tumoral NK-Cell Activity in Hepatocellular Carcinoma
Source: Cells. 2021 Jun 2;10(6):1369. doi: 10.3390/cells10061369 (PMC8227986; doi:10.3390/cells10061369)
Supplement: Supplementary file 1 [file cells-10-01369-s001.zip › cells-1180209-supplementary.pdf]

Adaptive subsets limit the anti-tumoral NK-cell activity in Hepatocellular Carcinoma

Charlotte Rennert*[1], Catrin Tauber*[1,2], Pia Fehrenbach[1,2], Kathrin Heim[1,2], Dominik Bettinger[1], Oezlem Sogukpinar[1], Anita Schuch[1,2], Britta Franziska Zecher[1,3], Bertram Bengsch[1], Sven A. Lang [4,5], Peter Bronsert[6,7], Niklas K. Björkström[8], Stefan Fichtner-Feigl[4], Michael Schultheiss[1], Robert Thimme[1] and Maike Hofmann[1]

* These authors contributed equally

Table of contents

Supplementary materials and methods 2

Assessment of NK-cell function 2

Multiparametric flow cytometry 3

Dimensionality reduction of multiparametric flow cytometry data 3

Supplementary figures 4

SI Figure 1. Increased frequency of FcεRIγ⁻ adaptive NK cells in HCMV⁺ HCC patients. 4

SI Figure 2. Adaptive NK-cell profile is comparable between HCC patients and control cohorts. 5

SI Figure 3. Adaptive NK-cell repertoire in blood and tissue of HCC patients. 6

SI Figure 4. $CD56^{dim}$ NK cells do not express tissue residency markers in matched tumor/non-tumor HCC tissue. 7

SI Figure 5. $CD56^{dim}$ NK cells do not express tissue residency markers in liver tissue. 8

SI Figure 6. MIP-1ß production by $Fc\varepsilon RI\gamma^-$ adaptive and $Fc\varepsilon RI\gamma^+$ conventional NK cells in HCC patients. 9

SI Figure 7. IFNγ production by $Fc\varepsilon RI\gamma^-$ adaptive and $Fc\varepsilon RI\gamma^+$ conventional NK cells in HCC patients. 10

SI Figure 8. Negative controls for functional NK-cell studies. 10

SI Figure 9. NCR and NKG2A expression of adaptive NK cells in HCC patients. 11

SI Figure 10. Conserved cytokine production of $CD56^{dim}$ NK-cell subpopulations 12

SI Figure 11. Frequency of $Fc\varepsilon RI\gamma^-$ adaptive NK cells do not correlate with AFP values 13

SI Figure 12. Conserved functional profile of $Fc\varepsilon RI\gamma^-$ adaptive $CD56^{dim}$ NK cells between $HCMV^+$ HBV and $HCMV^+$ HBV-associated HCC patients. 13

Supplementary Tables 14

SI Table 1. Study cohort of HCC patients (PBMCs) 14

SI Table 2. Study cohort of healthy donors (HD). 19

SI Table 3. Study cohort of patients with liver cirrhosis. 20

SI Table 4. Study cohort of patients with chronic HBV infection (HBV). 21

SI Table 5. Study cohort of liver and tumor samples from the hepatologic and gastroenterologic biobank of the University hospital Freiburg. 22

SI Table 6. Study cohort of liver samples obtained from the Karolinska Institute, Sweden.23

Supplementary materials and methods

Assessment of NK-cell function

For cytokine stimulation, 10^6 PBMCs were incubated overnight (o/n) at 37°C in IMDM culture medium (IMDM (Gibco-ThermoFisher), 10% fetal bovine serum, 1% penicillin-streptomycin and 50µM β-mercaptoethanol (Sigma-Aldrich) in the presence of IL-12 and IL-18 (IL-12 10ng/mL, Sigma-Aldrich; IL-18 5ng/mL, MBL) or without cytokines as negative control. Anti-CD107a mAb and anti-CD56 mAb were added directly. 0.96nmol/ml Brefeldin A (BD Biosciences) and 0.25nmol/ml Monensin (BD Biosciences) were added for the last four hours of incubation.

For co-culture assays with the cell lines K562, HuH7 and HepG2 cells, NK cells were isolated from PBMCs using MACS cell separation technology (NK cell isolation kit, Miltenyi Biotech) according to the manufacturer's instructions. Isolated NK cells and target cells or NK cells alone as a negative control were incubated in IMDM culture medium in the presence of anti-CD107a mAb and anti-CD56 mAb for five hours at 37°C. 0.96nmol/ml Brefeldin A (BD Biosciences) and 0.25nmol/ml Monensin (BD Biosciences) were added for the last four hours of stimulation. The effector to target ratio was 1:5 for stimulation with HuH7 and HepG2 cell lines and 1:10 for stimulation with K562 cell line.

For CD16-crosslinking, high-binding 96-well flat-bottom plates (Sigma-Aldrich) were coated o/n at 4°C with anti-CD16 pure IgG (10µg/ml in PBS, BD Bioscience) or PBS only, as negative control. After extensive washing, 10^6 PBMCs in IMDM culture medium in the presence of anti-CD107a mAb and anti-CD56 mAb were added to the wells and incubated for five hours at 37°C and 5% CO_2. 0.96nmol/ml Brefeldin A (BD Biosciences) and 0.25nmol/ml Monensin (BD Biosciences) were added for the last four hours of incubation (37°C and 5% CO_2).

For stimulation with autologous activated $CD8^+$ T cells, $CD8^+$ T cells were isolated from PBMCs using MACS cell separation technology ($CD8^+$ T cell isolation kit, Miltenyi Biotech) according to the manufacturer's instructions. Isolated $CD8^+$ T cells were stimulated for 60h with ImmunoCult™ Human CD3/CD28 T Cell Activator (Stemcell Technologies) in complete RPMI culture medium. Activated $CD8^+$ T cells and freshly isolated autologous NK cells were co-incubated at a T-cell/NK-cell ratio of 1:10 for 5h in the presence of anti-CD107a mAb and anti-CD56 mAb. 0.96 nmol/ml Brefeldin A (BD Biosciences) and 0.25 nmol/ml Monensin (BD Biosciences) were added for the last four hours of stimulation. NK cells were isolated from PBMCs using MACS cell separation technology (NK cell isolation kit, Miltenyi Biotech) according to the manufacturer's instructions.

Subsequently, surface and intracellular stainings were performed.

Multiparametric flow cytometry

For flow cytometry the following antibodies were used: anti-CD3 (SK7), anti-CD4 (SK3), anti-CD8 (RPA-T8), anti-CD16 (3G8), anti-CD49a (SR84), anti-CD56 (B159), anti-CD57 (NK-1), anti-CD107a (H4A3), anti-CXCR6 (13B1E5), anti-PD1 (EH12.1), anti-PLZF (R17-809), anti-NKp46 (9E2/NKp46), anti-NKp30 (p30-15), anti-CD2 (RPA-2.10) (BD Biosciencesanti-CD3 (SK7 and UCHT1), anti-CD45 (HI30), anti-CD56 (5.1H11), anti-CD57 (QA17A04), anti-CXCR6 (K041E5), anti-IFN-γ (4S.B3), anti-NKG2D (1D11), anti-TIGIT (A15153G) anti-TNF (Mab11), anti-Siglec7 (6-434), anti-CD7 (CD7-6B7), anti-CXCR3 (G025H7) (BioLegend), anti-CD14 (61D3), anti-CD19 (HiB19), anti-CD69 (FN50), anti-Helios (22F6), anti-MIP-1β (FL34Z3L), anti-Syk (4D10.1), anti-TIGIT (MBSA43), anti-CX3CR1 (2A9-1) (eBioscience-Thermo) anti-FcεRIγ (polyclonal) (Millipore), anti-NKG2A (131411) and anti-NKG2C (134591) (R&D Systems).

Fixable Viability Dyes (eFluor780, eBioscience-Thermo Fisher) was used for live/dead discrimination. FoxP3/Transcription Factor Staining Buffer Set (eBioscience-Thermo Fisher) was applied according to the manufacturer's instructions for intranuclear staining. Cells were fixed with paraformaldehyde and analyzed using FACSCanto II or LSRFortessa (BD Biosciences).

Dimensionality reduction of multiparametric flow cytometry data

The visualization of multiparametric flow cytometry data was done with R using the Bioconductor (CATALYST package (Crowell H, Zanotelli V, Chevrier S, Robinson M (2020). CATALYST: Cytometry dATa anALYSis Tools. R package version 1.12.2, https://github.com/HelenaLC/CATALYST). The analyses were performed on gated FcεRIγ^{-} CD56^{dim} NK cells and included the markers NKG2C, PLZF, CD57, Helios, CD16, PD-1, TIGIT and NKG2D. Down sampling of cells to the group comprising the lowest cell count or to 3000 cells was performed prior to dimensionality reduction in order to facilitate the visualization of different samples. Marker intensities were transformed by arcsinh (inverse hyperbolic sine) with a cofactor of 150. Dimensionality reduction on the transformed data was achieved by t-distributed stochastic neighbor embedding (t-SNE) using the CATALYST package functions runDR with default parameters.

Supplementary figures

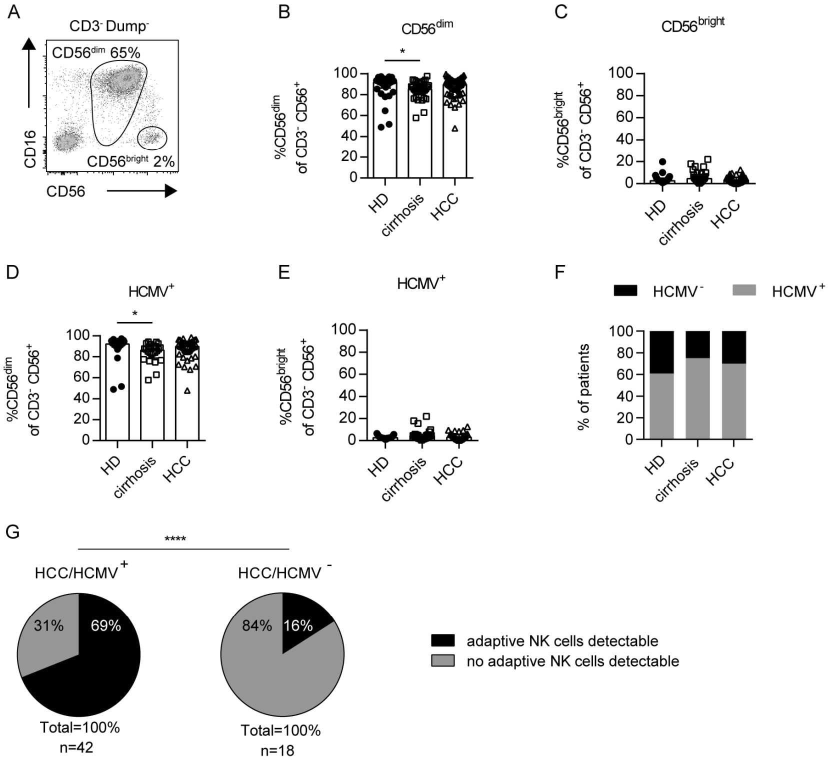

Figure S1. Increased frequency of FcεRIγ$^-$ <u>adaptive</u> NK cells in HCMV$^+$ HCC patients.
Gating strategy (A). Frequency of CD56^{dim} (B) and CD56^{bright} (C) NK cells of CD3$^-$CD56$^+$ lymphocytes in the peripheral blood of HCC patients (n=60), HD (n=33) and patients with liver cirrhosis (n=33). Frequency of CD56^{dim} (D) and CD56^{bright} (E) NK cells in HCMV$^+$ individuals (HCC n=42, HD n=19, patients with liver cirrhosis n=24). Percentage of HCMV$^+$ (grey) and HCMV$^-$ (black) patients (F, n equal to B and C). Pie charts depicting presence of FcεRIγ$^-$ adaptive CD56^{dim} NK cells (black) and absence of FcεRIγ$^-$ CD56^{dim} adaptive NK cells (grey) in HCMV$^+$ (left) and HCMV$^-$ (right) HCC patients. Patients with adaptive NK cells are defined as >10% FcεRIγ$^-$ adaptive of all CD56^{dim} NK cells (G). Each dot represents an individual. Bars indicate the median with IQR. Statistical significance was assessed by using Kruskal-Wallis test (B-E) and with parts of whole analysis (binomial test, G). HCMV: human cytomegalovirus, HCC: patients with hepatocellular carcinoma, HD: healthy donors, cirrhosis: patients with liver cirrhosis. Dump$^-$ cells exclude doublets, dead cells, CD14$^+$ and CD19$^+$ cells.

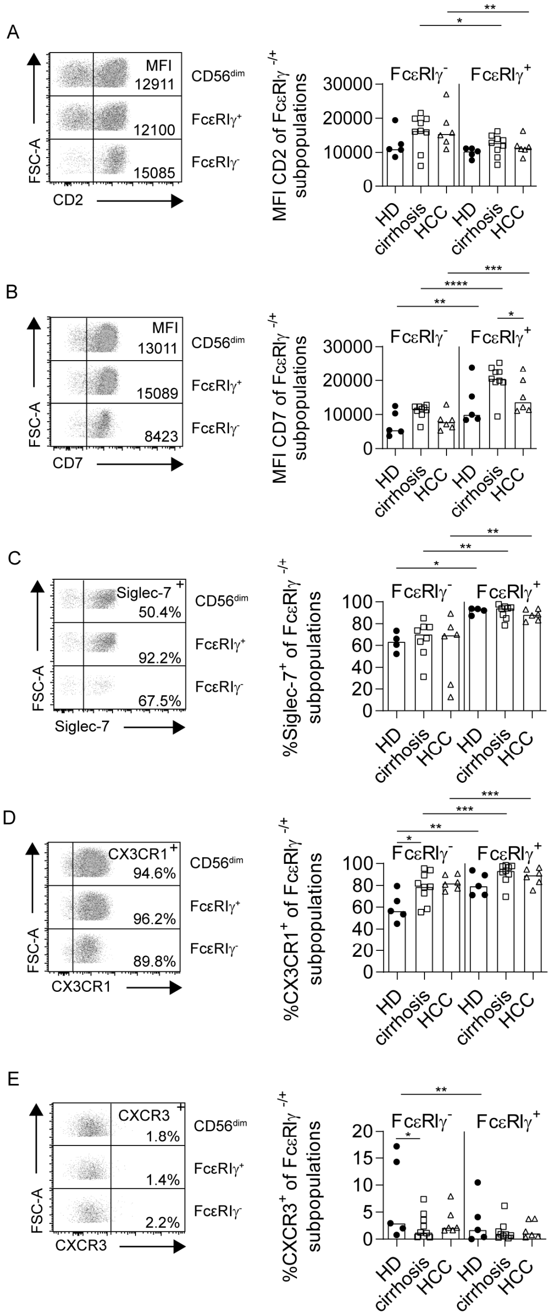

Figure S2. Adaptive NK-cell profile is comparable between HCC patients and control cohorts. CD2 (A), CD7 (B), Siglec-7 (C), CX3CR1 (D) and CXCR3 (E) expression on FcεRIγ- subpopulations in HCMV+ HD (n=5 in A, B, D and E, n=4 in C), HCMV+ patients with liver cirrhosis (n=9 in A, B, D and E, n=8 in C) and HCMV+ HCC patients (n=6). Each point represents a single patient with more than 10% adaptive FcεRIγ- of CD56dim NK cells. Bars indicate median. Statistical significance was tested using mixed-effect analysis. HCMV: human cytomegalovirus, HCC: patients with hepatocellular carcinoma, HD: healthy donors, cirrhosis: patients with liver cirrhosis.

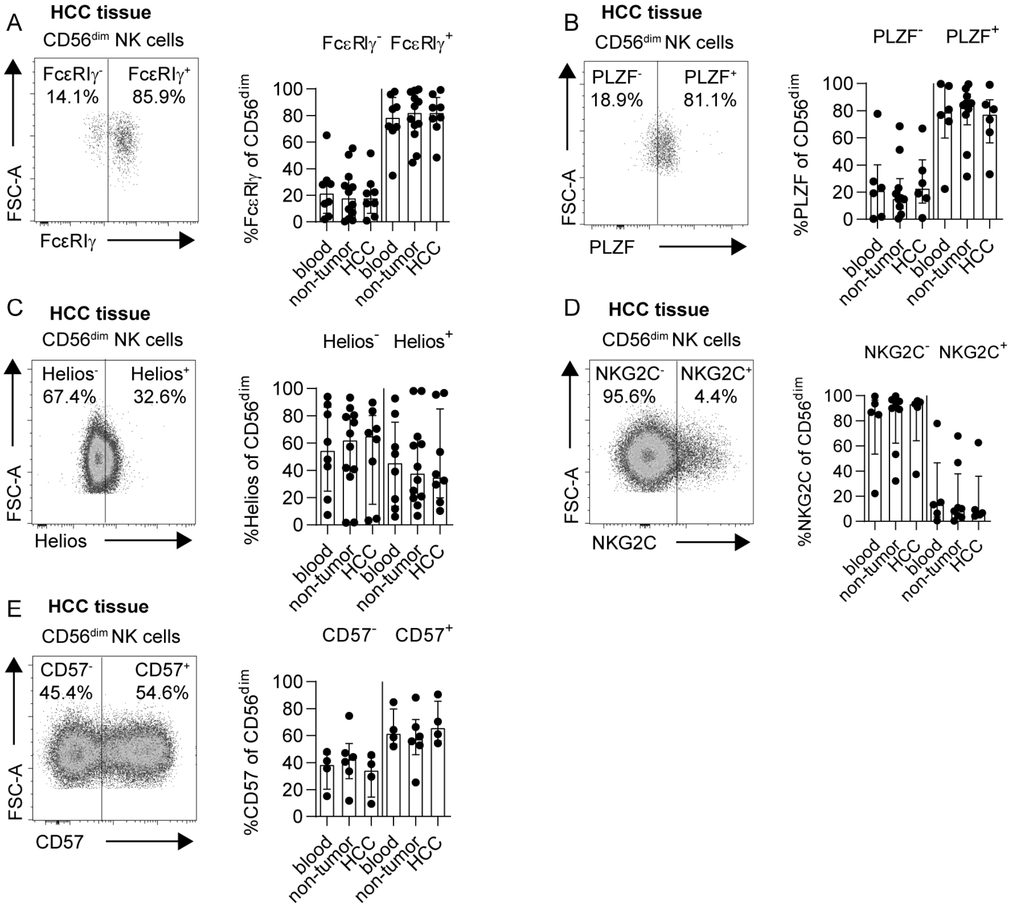

Figure S3. Adaptive NK-cell repertoire in blood and tissue of HCC patients.

FcεRIγ (A), PLZF (B), Helios (C), NKG2C (D) and CD57 (E) expression on $CD56^{dim}$ NK cells in HCC tissue (n=8 in A, and C, n=6 in B, n=5 in D, n=4 in E), adjacent non-tumoral liver tissue (n= 12 in A and C, n=10 in B, n=8 in D, n=6 in E) and matched blood samples (n=8 in A, and C, n=6 in B, n=5 in D, n=4 in E). Bars indicate median with IQR. Statistical significance for cohort comparison was tested using Kruskal-Wallis test. HCC: hepatocellular carcinoma tumor tissue.

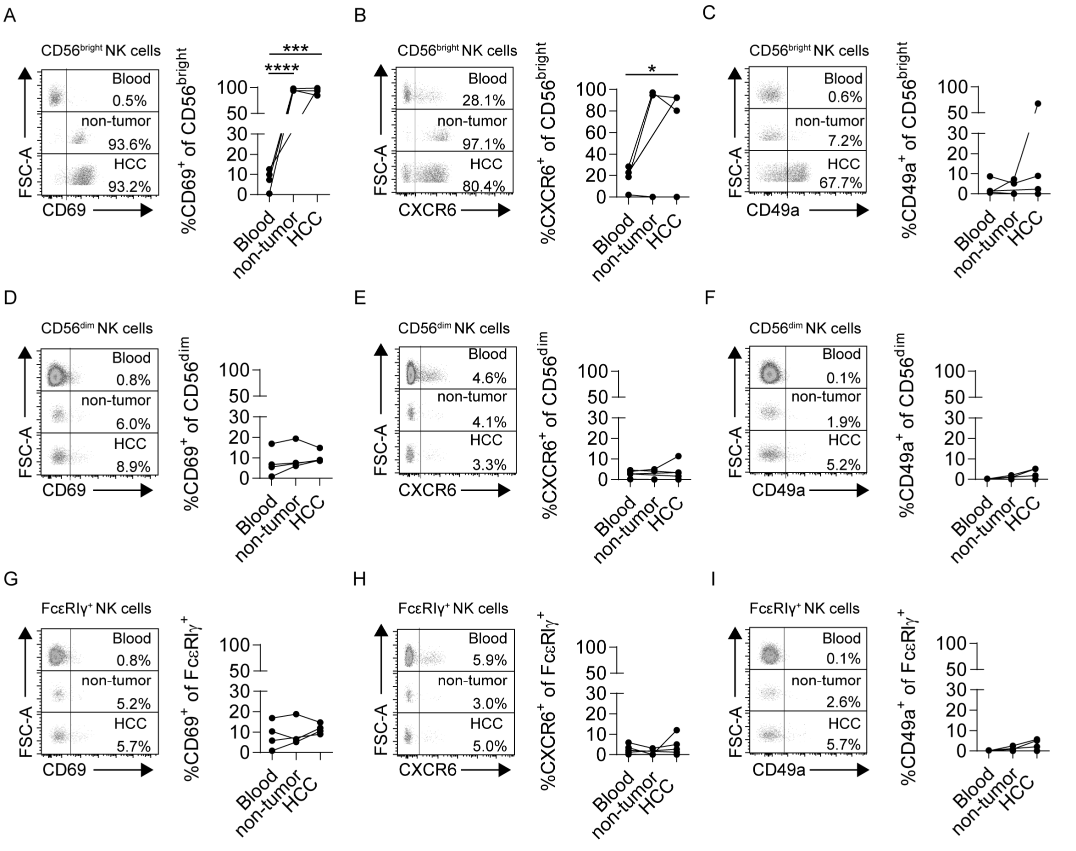

Figure S4. CD56^{dim} NK cells do not express tissue residency markers in matched tumor/non-tumor HCC tissue.

CD69, CXCR6 and CD49a expression on CD56^{bright} (A-C) CD56^{dim} (D-F) and FcεRIγ^{+} conventional (G-I) CD56^{dim} NK cells in blood (n=4 for A, C, D, F, G and I, n=5 for B, E and H), adjacent non-tumor (n=3) and HCC tumor tissue (n equal to blood) of HCC patients. Each point represents a single HCMV^{+} patient and the samples from one patient are connected by the line. Statistical analysis was performed with mixed-effect analysis. HCC: hepatocellular carcinoma tumor tissue.

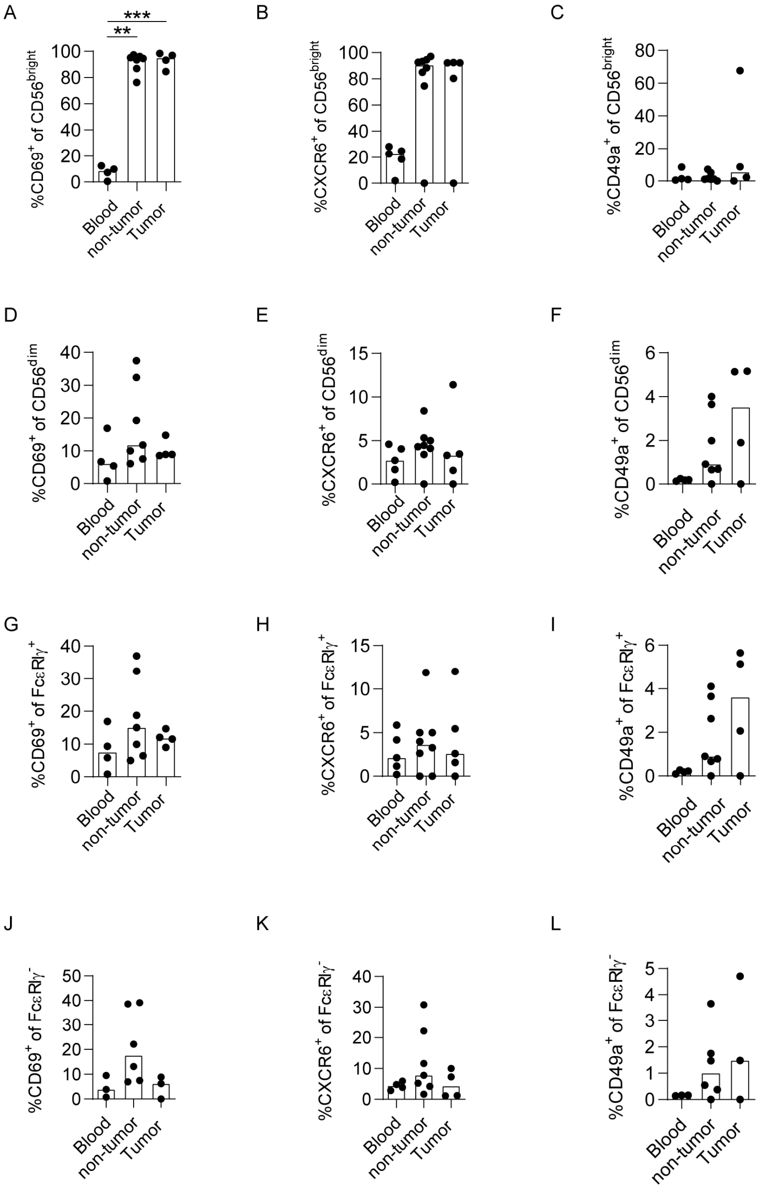

Figure S5. CD56^{dim} NK cells do not express tissue residency markers in liver tissue.
CD69, CXCR6 and CD49a expression on CD56^{bright} (A-C) CD56^{dim} (D-F) and FcεRIγ^{+} (G-I) and FcεRIγ- (J-L) CD56^{dim} NK cells in blood (n=4 for A,C,D,F,G, K and I, n=5 for B,E and H, n=3 for J and L), non-tumor liver tissue (n=8 for B,E and H, n=7 for A,C,D,F,G,I and K, n=6 for J and L) and HCC tumor tissue (n equal to blood). Each point represents a single HCMV^{+} donor. Statistical analysis was performed with mixed-effect analysis. HCC: Hepatocellular carcinoma, HCMV: human cytomegalovirus.

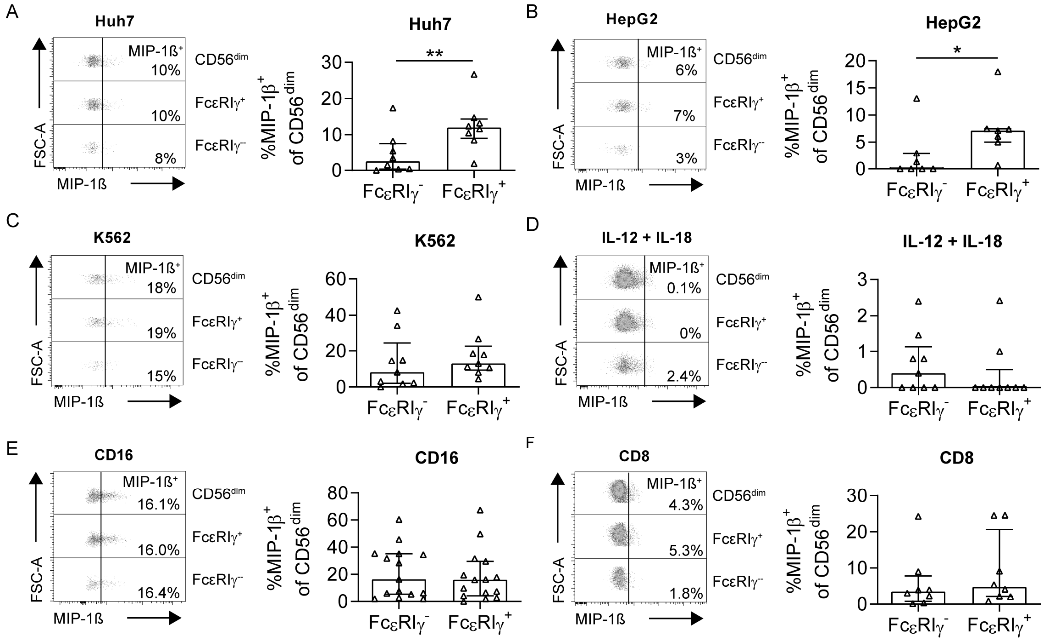

Figure S6. MIP-1ß production by FcεRIγ- adaptive and FcεRIγ+ conventional NK cells in HCC patients.

MIP-1ß expression of CD56dim NK cells following stimulation with Huh7 (A, n=8), HepG2 (B, n=7) or K562 (C, n=9) cell lines for 5h, cytokine stimulation with IL-12 and IL-18 overnight (D, n=9), CD16 crosslink (E, n=15) or stimulation with autologous activated CD8+ T cells for 5h (F, n=8) in HCMV+ HCC patients. Each dot represents an individual with more than 10% FcεRIγ- adaptive CD56dim NK cells. Bars indicate median with IQR. Statistical significance was tested using paired two-tailed Wilcoxon test.

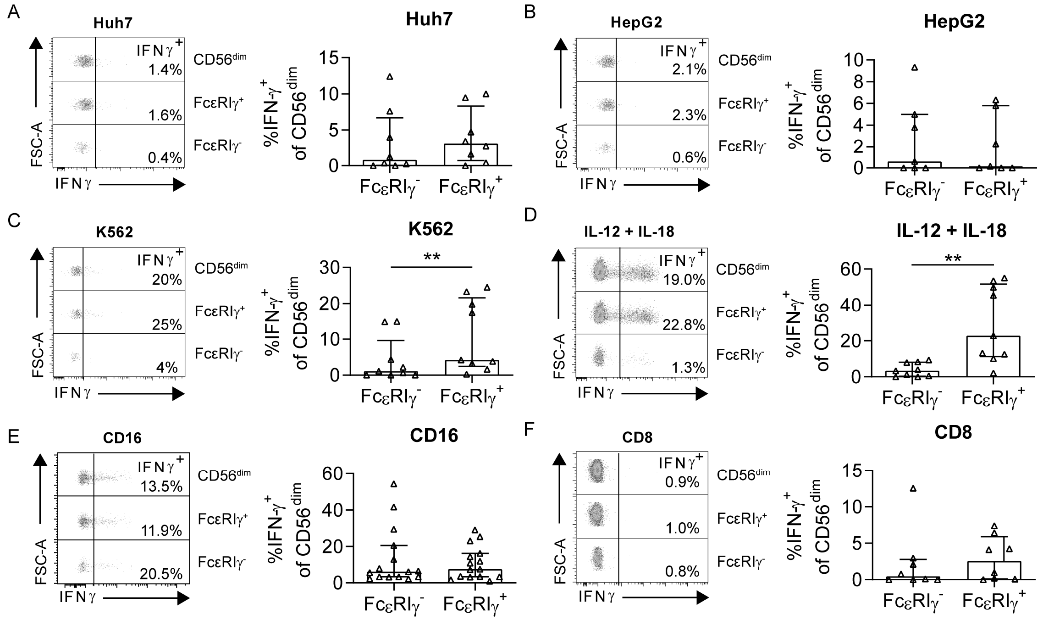

Figure S7. IFNγ production by FcεRIγ$^-$ adaptive and FcεRIγ$^+$ conventional NK cells in HCC patients.

IFNγ expression of CD56^{dim} NK cells following stimulation with Huh7 (A, n=8), HepG2 (B, n=7) or K562 (C, n=9) cell lines for 5h, cytokine stimulation with IL-12 and IL-18 overnight (D, n=9), CD16 crosslink (E, n=15) or stimulation with autologous activated CD8$^+$ T cells for 5h (F, n=8) in HCMV$^+$ HCC patients. Each dot represents an individual with more than 10% FcεRIγ$^-$ adaptive CD56^{dim} NK cells. Bars indicate median with IQR. Statistical significance was tested using paired two-tailed Wilcoxon test.

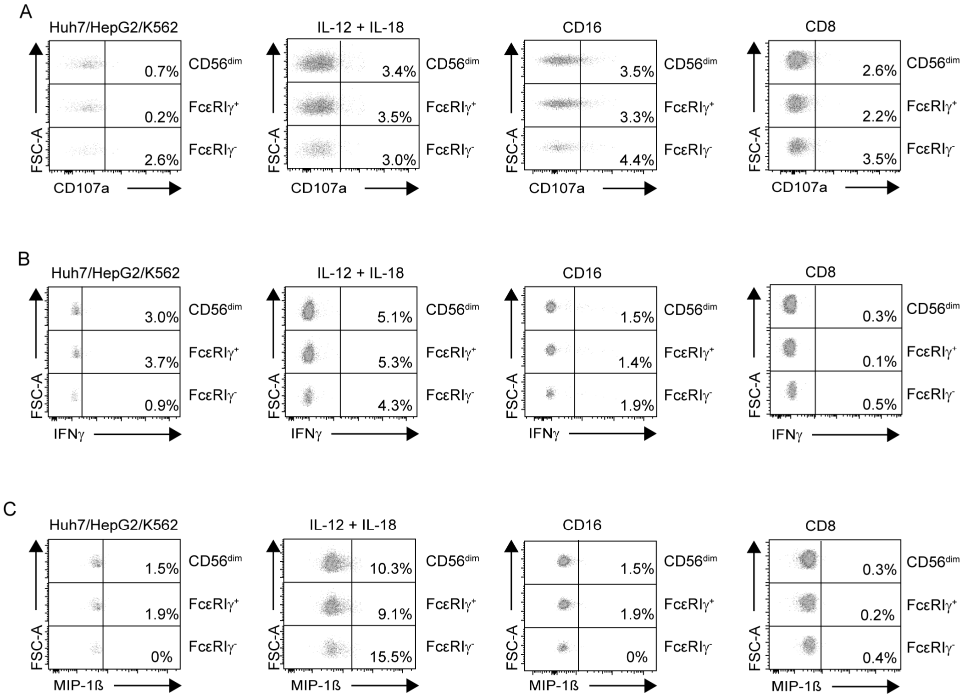

Figure S8. Negative controls for functional NK-cell studies.

CD107a (A), IFNγ (B) and MIP-1ß (C) expression of CD56^{dim} NK cells, FcεRIγ$^+$ and FcεRIγ$^-$ CD56^{dim} NK cells without stimulation.

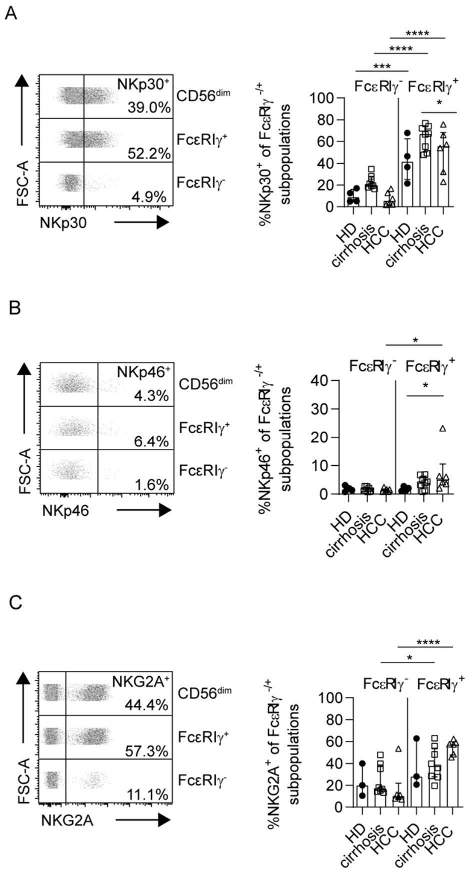

Figure S9. NCR and NKG2A expression of adaptive NK cells in HCC patients.

NKp30 (A), NKp46 (B) and NKG2A (C) expression of FcεRIγ-subpopulations in HCC patients (n=6), HD (n=4 in A and B, n=3 in C) and patients with liver cirrhosis (n=8). Each dot represents an HCMV⁺ individual with more than 10% adaptive FcεRIγ⁻ CD56^dim NK cells. Bars indicate median with IQR. Statistical significance was tested by using mixed-effect analysis. HCC: patients with hepatocellular carcinoma, HD: healthy donors, cirrhosis: patients with liver cirrhosis.

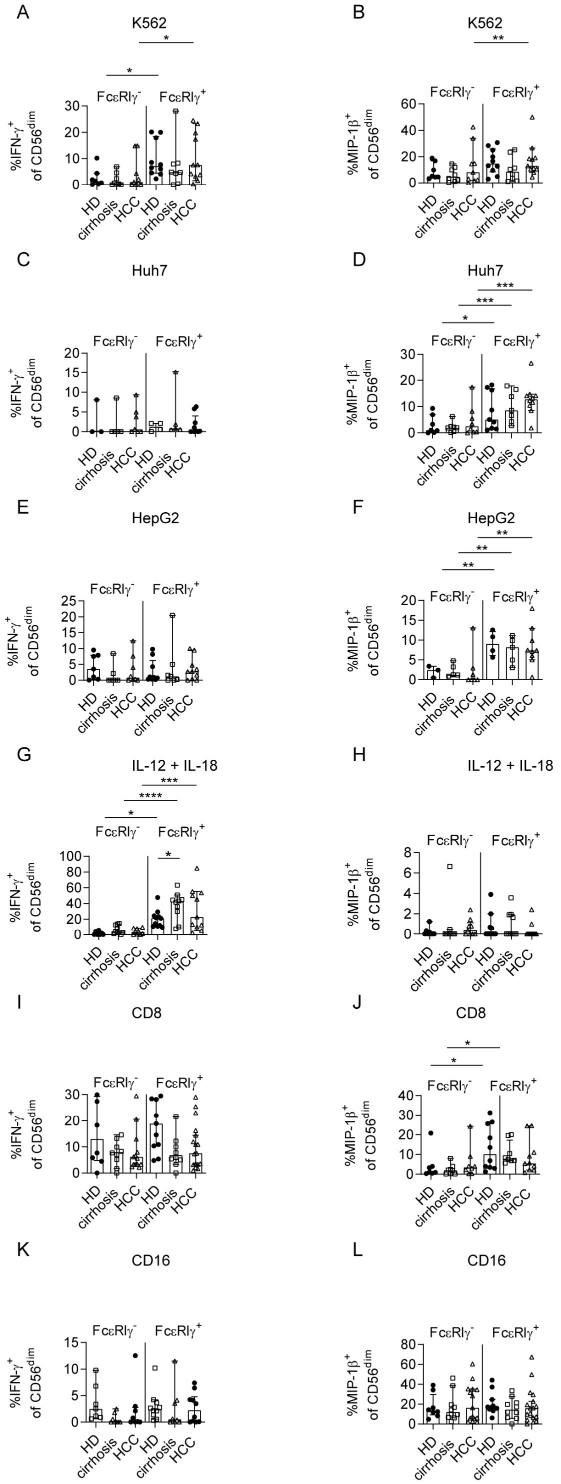

Figure S10. Conserved cytokine production of CD56^{dim} NK-cell subpopulations.

IFNγ (left) and MIP-1ß (right) expression of CD56^{dim} NK cells following stimulation with K562 (A,B, HCC FcεRIγ$^-$ n=9, FcεRIγ$^+$ n=11, HD FcεRIγ$^-$ n=7, FcεRIγ$^+$ n=10, cirrhosis n=8), Huh7 (C,D, HCC FcεRIγ$^-$ n=8, FcεRIγ$^+$ n=10, HD FcεRIγ$^-$ n=7, FcεRIγ$^+$ n=9, cirrhosis n=7), and HepG2 cell lines (E,F, HCC FcεRIγ$^-$ n=7, FcεRIγ$^+$ n=9, HD FcεRIγ$^-$ n=3, FcεRIγ$^+$ n=4, cirrhosis n=5) for 5h, cytokine stimulation with IL-12 and IL-18 overnight (G,H, HCC FcεRIγ$^-$ n=9, FcεRIγ$^+$ n=11, HD FcεRIγ$^-$ n=8, FcεRIγ$^+$ n=11, cirrhosis n=10), stimulation with autologous activated CD8$^+$ T cells for 5h (I,J, HCC FcεRIγ$^-$ n=8, FcεRIγ$^+$ n=10, HD

FcεRIγ⁻ n=7, FcεRIγ⁺ n=10, cirrhosis n=8) or CD16 crosslink (K,L, HCC FcεRIγ⁻ n=15, FcεRIγ⁺ n=17, HD FcεRIγ⁻ n=8, FcεRIγ⁺ n=11, cirrhosis n=10) in HCMV⁺ HCC patients and control cohorts. Each dot represents an individual with more than 10% FcεRIγ⁻ adaptive CD56^{dim} NK cells. Bars indicate median with IQR. Statistical significance was tested using paired mixed-effect analysis. HCMV: human cytomegalovirus, HCC: patients with hepatocellular carcinoma, HD: healthy donors, cirrhosis: patients with liver cirrhosis.

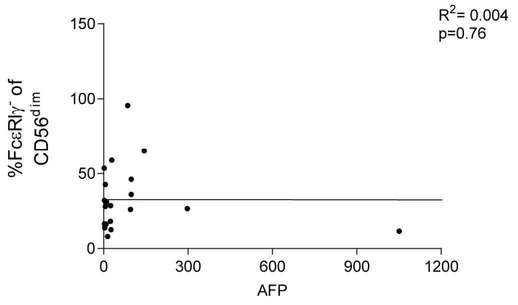

Figure S11. Frequency of FcεRIγ⁻ adaptive NK cells do not correlate with AFP values.
Correlation analysis of AFP value and frequencies of FcεRIγ⁻ CD56^{dim} NK cells in HCMV⁺ HCC patients (n=20). Statistical analysis was performed with linear regression analysis. Patients with less than 10% FcεRIγ⁻ adaptive CD56^{dim} NK cells were excluded. HCMV: human cytomegalovirus, HCC: patients with hepatocellular carcinoma.

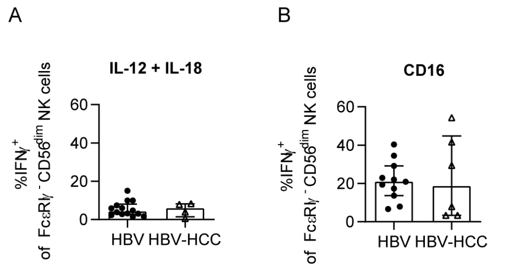

Figure S12. Conserved functional profile of FcεRIγ⁻ adaptive CD56^{dim} NK cells between HCMV⁺ HBV and HCMV⁺ HBV-associated HCC patients.
IFNγ production of CD56^{dim} NK cells following cytokine stimulation with IL-12 and IL-18 overnight (A) or CD16 crosslink (B) in HCMV⁺ chronic HBV (n=14 in A, n=11 in B) and HBV-associated HCC patients (n=4 in A, n=6 in B). Each dot represents an individual with more than 10% FcεRIγ⁻ adaptive CD56^{dim} NK cells. Bars indicate median with IQR. Statistical analysis was performed with Mann-Whitney test. HBV: hepatitis B virus, HCC: patients with hepatocellular carcinoma, HCMV: human cytomegalovirus.

Supplementary Tables

Table S1. Study cohort of HCC patients (PBMCs).

M: male, F: female, AIH: autoimmune hepatitis, ASH: alcohol induced steatohepatis, HBV: hepatitis B virus, HCV: hepatitis C virus, HDV: hepatitis D virus, HFE: hemochromatosis, NASH: non-alcoholic steatohepatis, n.d.: not determined, neg: negative, pos: positive, SI: supplementary information.

If not stated otherwise each donor is included in every subsection of the figures.

* liver and/or tumor tissue available, see SI table 6 for details.

Patient ID	Age [years]	Sex	Etiology	Child score	BCLC score	AFP [ng/ml]	GPT [U/l]	GOT [U/l]	HCMV sero-status	Treatment prior inclusion	Inclusion in figures
HCC#1	78	M	ASH	B	B	n.d.	n.d.	n.d.	pos	no	1, 3 A-E, 4 A/C/E, 5 A-E, 6 A-D, SI 1 B-G, SI 6 A-E, SI 7 A-E, SI 10 A-H/K/L
HCC#2	45	M	HBV	A	C	n.d.	296	235	pos	no	1 A-F, I, 4 A/C, 5, 6 C/D, SI 1 B-G, SI 10
HCC#3	59	F	HBV, HCV, ASH	A	B	15	144	145	pos	no	1 A/C-I, 3, 4 C/E, 5, 6 A-D/F/G, SI 1 B-G, SI 6, SI 7, SI 10, SI 11, SI 12
HCC#4	67	M	HBV	no cirrhosis	B	n.d.	75	67	pos	no	1, 3, 4 C/E, 5, 6 B-G, SI 1 B-G, SI 6, SI 7, SI 10, SI 11, SI 12
HCC#5	59	M	ASH	A	B	24	71	74	pos	no	1 A/C-I, 3, 4 C/E, 5, 6 A/C/D, SI 1 B-G, SI 6, SI 7, SI 10, SI 11
HCC#6	55	M	HCV	B	B	26537	81	n.d.	pos	no	1, 4 C/E, 5, 6 D, SI 1 B-G, SI 10, SI 11
HCC#7	55	M	HCV	A	B	9	99	95	pos	no	1, 3, 4 C/E, 5, 6 A/B/D, SI 1 B-G, SI 6, SI 7, SI 10, SI 11
HCC#8	49	M	HCV, ASH	A	0	5	216	103	pos	no	1, 3, 4 C/E, 5, 6 A/B/D, SI 1 B-G, SI 6, SI 7, SI 10, SI 11
HCC#9	73	M	HBV, HCV	A	A	29	47	85	pos	no	1, 3 B/E, 4 C/E, 5 B/E, 6 A/B/D/F/G, SI 1 B-G, SI 2, SI 6 B/E, SI 7 B/E, SI 9, SI 10 E/F/K/L, SI 11, SI 12 B

Patient ID	Age [years]	Sex	Etiology	Child score	BCLC score	AFP [ng/ml]	GPT [U/l]	GOT [U/l]	HCMV sero-status	Treatment prior inclusion	Inclusion in figures
HCC#10	71	M	ASH	A	B	n.d.	43	64	neg	resection, TACE	SI 1 B/C/F/G
HCC#11	67	M	NASH	A	C	n.d.	35	45	pos	resection, TACE,	1 A/C-I, SI 1 B-G
HCC#12	47	M	HCV, ASH	B	A	3	31	34	neg	resection	SI 1 B/C/F/G
HCC#13	62	M	ASH	C	B	n.d.	n.d.	168	pos	TACE, SIRT	1, 4 A/C/E, 6 D, SI 1 B-G
HCC#14	52	F	HCV	A	B	9694	32	32	pos	TACE	1, 4 A/C/E, 6 D, SI 1 B-G
HCC#15	64	M	HCV	A	A	5	60	65	neg	resection,	SI 1 B/C/F/G
HCC#16	54	M	HCV	A	B	n.d.	n.d.	n.d.	pos	3xTACE	1, 4 A/C/E, 6 D, SI 1 B-G
HCC#17	79	M	ASH	A	B	3	40	41	neg	TACE	SI 1 B/C/F/G
HCC#18	78	M	ASH	A	C	2	30	52	pos	TACE	1, 4 A/C/E, 6 D, SI 1 B-G
HCC#19	67	M	HCV	A	A	n.d.	101	97	pos	no	1, 4 A/C/E, 6 C/D, SI 1 B-G
HCC#20	54	F	NASH	no cirrhosisis	B	4	62	47	neg	resection	SI 1 B/C/F/G
HCC#21	84	F	NASH	A	A	n.d.	52	56	neg	no	SI 1 B/C/F/G
HCC#22	80	M	NASH	A or no cirrhosis	B	n.d.	64	46	neg	no	SI 1 B/C/F/G
HCC#23	62	M	ASH	A	C	1051	15	n.d.	pos	resection	1, 3 E, 4 A/C/E, 5 E, 6 A/B/D, SI 1 B-G, SI 2, SI 6 E, SI 7 E, SI 10 K/L, SI 9, SI 10
HCC#24	77	M	ASH	A	B	62	73	n.d.	neg	no	SI 1 B/C/F/G
HCC#25	62	M	n.d.	B	A	13	28	59	neg	no	SI 1 B/C/F/G
HCC#26	81	F	NASH	no cirrhosis	A	6	32	34	neg	no	SI 1 B/C/F/G

Patient ID	Age [years]	Sex	Etiology	Child score	BCLC score	AFP [ng/ml]	GPT [U/l]	GOT [U/l]	HCMV sero-status	Treatment prior inclusion	Inclusion in figures
HCC#27	79	M	ASH, NASH	A	B	71	n.d.	n.d.	neg	3xTACE, SBRT	SI 1 B/C/F/G
HCC#28	69	M	HBV	A	B	n.d.	46	57	neg	7xTACE	SI 1 B/C/F/G
HCC#29	74	M	HBV	B	C	n.d.	90	101	neg	resection,	SI 1 B/C/F/G
HCC#30	70	M	ASH	A	A	1616	50	87	pos	no	1, 4 A/C/E, 6 C/D, SI 1 B-G
HCC#31	60	M	ASH	B	A	10	n.d.	n.d.	pos	no	1, 3 E, 4 A/C/E, 5 E, 6 A-D, SI 1 B-G, SI 2, SI 6 E, SI 7 E, SI 9, SI 10 K/L, SI 11
HCC#32	58	M	HCV	B	A	11	34	n.d.	neg	TACE	SI 1 B/C/F/G
HCC#33	53	M	ASH, AIH,	C	A	466	73	122	neg	no	SI 1 B/C/F/G
HCC#34	65	F	ASH	A	A	5	14	20	neg	TACE	SI 1 B/C/F/G
HCC#35	61	M	NASH	C	B	15	51	99	pos	TACE	1, 4 A/C/E, SI 1 B-G
HCC#36	55	M	HBV/HDV	B	B	n.d.	67	112	pos	no	1, 3 E, 4 A/C/E, 5 E, 6, SI 1 B-G, SI 2, SI 6 E, SI 7 E, SI 9, SI 10 K/L, SI 12 B
HCC#37	53	M	NASH	A	A	3	46	32	neg	TACE+RFTA	SI 1 B/C/F/G
HCC#38	80	M	HBV	B	B	>60500	58	95	pos	no	1, 4 A/C/E, 5 E, 6 A/B/D-G, SI 1 B-G, SI 9, SI 11
HCC#39	71	M	HBV/HDV	no cirrhosis	B	2	n.d.	n.d.	neg	TACE	SI 1 B/C/F/G
HCC#40	61	M	ASH	A	A	6	74	47	pos	resection	1 A, C-I, 6 A/B/D, SI 1 B-G, SI 11
HCC#41	64	F	HBV	no cirrhosis	A	27	23	34	pos	resection	1, A, C-I, 6 B/D/F/G, SI 1 B-G, SI 11

Patient ID	Age [years]	Sex	Etiology	Child score	BCLC score	AFP [ng/ml]	GPT [U/l]	GOT [U/l]	HCMV sero-status	Treatment prior inclusion	Inclusion in figures
HCC#42	75	M	ASH	A	A	3	65	79	pos	TACE	1 A, C-H, 6 A/B, SI 1 B-G, SI 12
HCC#43	68	M	HCV	A	A	n.d.	n.d.	n.d.	pos	2xTACE	1, 3 E, 4 A/C/E, 5 E, 6 A/B/D, SI 1 B-G, SI 2, SI 6 E, SI 7 E, SI 9, SI 10 K/L, SI 11
HCC#44	71	M	HCV, ASH	A	A	3	65	79	pos	resection	1, 3 E, 4 A/C/E, 5 E, 6 A/B/D, SI 1 B-G, SI 2, SI 6 E, SI 7 E, SI 9, SI 10 K/L, SI 11
HCC#45	67	M	HBV	A	B	n.d.	n.d.	n.d.	pos	4xTACE, SBRT	1, 4 A/C/E, 5 E, 6 A/B/D/E-G, SI 1 B-G, SI 9, SI 11
HCC#46	65	M	HFE	A	C	98	n.d.	n.d.	pos	2xTACE	1, 4 A/C/E, 5 E, 6 A/B, SI 1 B-G, SI 9, SI 11
HCC#47	76	F	AIH	A	A	422	40	69	pos	resection	1, 4 C, SI 1 B-G
HCC#48	65	M	HBV, HCV	B	B	298	189	209	pos	no	1, 3 A/C-F, 4 C, 5 A/C-F, 6 A/B/D-G, SI 1 B-F, SI 6 A/C-F, SI 7 A/C-F, SI 10 A/G-L, SI 11, SI 12
HCC#49	81	M	ASH	A	C	n.d.	n.d.	n.d.	pos	no	1 A/C-I, 3 A/C-F, 4 C, 5 A/C-F, 6 A/B/D, SI 1 B-G, SI 6 A/C-F, SI 7 A/C-F, SI 10 A/G-L, SI 11
HCC#50	57	M	n.d.	A	B	9	n.d.	n.d.	pos	no	1, 4 C, SI 1 B-G

Patient ID	Age [years]	Sex	Etiology	Child score	BCLC score	AFP [ng/ml]	GPT [U/l]	GOT [U/l]	HCMV sero-status	Treatment prior inclusion	Inclusion in figures
HCC#51	70	M	HBV, ASH	A	C	7855	82	303	pos	no	1 A/C-I, 3 C-F, 4 C/E, 5 A/D-F, 6 A/B/D-G, SI 1 B-G, SI 6 C-F, SI 7 C-F, SI 10 A/B/G-L, SI 11, SI 12
HCC#52	76	M	HCV	A	A	226	20	n.d.	pos	no	1, 4 C/E, SI 1 B-G
HCC#53*	65	M	NASH	A	n.d.	n.d.	n.d.	n.d.	pos	no	1 A/C-F, 6 A, SI 1 B-G
HCC#54*	80	F	HCV	A	B	3692	86	132	pos	no	1 A/C-F, 6 A/B/D, SI 1 B-G, SI 11
HCC#55*	77	F	n.d.	n.d.	A	n.d.	n.d.	n.d.	pos	no	1 A/C-F, SI 1 B-G
HCC#56*	74	M	ASH	A	n.d.	6	40	41	pos	no	1 A/C-F, 6 A/B/D, SI 1 B-G, SI 11
HCC#57*	55	M	ASH	A	B	25	58	85	pos	no	1 A/C-E, 6 A/D, SI 1 B-G, SI 11
HCC#58*	71	M	ASH/NASH	A	A	144	26	41	pos	TACE	1 A/D, 4 B, 6 A/B/D, SI 1 B-F, SI 11
HCC#59*	79	M	steatosis hepatis	A	A	n.d.	n.d.	n.d.	pos	TACE, resection	1 A/D, 4 B, 6 A/B, SI 1 B-G, SI 11
HCC#60*	81	M	n.d.	no cirrhosis	B	6	27	21	pos	no	1 A/D, SI 1 B-G

Table S2. Study cohort of healthy donors (HD).
M: male, F: female, neg: negative, pos: positive, SI: Supplementary information.
If not stated otherwise each donor is included in every subsection of the figures.

Patient ID	Age [years]	Sex	CMV serostatus	Inclusion in figures
HD#1	55	M	neg	SI 1 B/C/F
HD#2	33	M	pos	1, 4 A/C/E, 5, 6 D, SI 1 B-F, SI 10
HD#3	26	F	pos	1, 4 A/C/E, 5, 6 D, SI 1 B-F, SI 10
HD#4	34	M	pos	1, 4 A/C/E, 5, 6 D, SI 1 B-F, SI 2, SI 9 A/B, SI 10
HD#5	45	F	pos	1, 4 C/E, 5, 6 D, SI 1 B-F, SI 10
HD#6	54	F	neg	SI 1 B/C/F
HD#7	56	M	neg	SI 1 B/C/F
HD#8	65	M	neg	SI 1 B/C/F
HD#9	55	F	neg	SI 1 B/C/F
HD#10	63	M	pos	1, 4 C/E, 5 A/C-F, 6 D, SI 1 B-F, SI 10 A-D, G-L
HD#11	61	M	neg	SI 1 B/C/F
HD#12	59	F	neg	SI 1 B/C/F
HD#13	54	F	pos	1, 4 C, 5 A/C-F, 6 D, SI 1 B-F SI 10 A-D, G-L
HD#14	79	F	pos	1, 4 C, 5 A/C-F, 6 D, SI 1 B-F, SI 2 A/B/D/E, SI 10 A-D, G-L
HD#15	85	F	pos	1, 4 C, 5 A/C-F, 6 D, SI 1 B-F, SI 10 A-D, G-L
HD#16	86	F	pos	1, 4 C/E, 6 D, SI 1 B-F
HD#17	55	M	pos	1, 4 C/E, 5 C-E, 6 D, SI 1 B-F, SI 2, SI 9, SI 10 C/DG/H/K/L
HD#18	82	F	pos	1, 4 C/E, 6 D, SI 1 B-F
HD#19	58	M	pos	1, 4 C/E, 6 D, SI 1 B-F
HD#20	54	F	pos	1 A-H, 4 C/E, 6 D, SI 1 B-F
HD#21	60	M	pos	1 A-H, 4 C/E, 5 A/D-F, 8 D, SI 1 B-F, SI 2, SI 9, SI 10 A/B/G-L
HD#22	54	M	pos	1, 4 C/E, 5 A/D-F, 8 D, SI 1 B-F, SI 10 A/B, G-L
HD#23	53	M	pos	1, 4 C/E, 6 D, SI 1 B-F
HD#24	57	M	pos	1 A/G/H, 6 D
HD#25	54	F	pos	1 A/G/H, 6 D
HD#26	54	F	pos	1 A/G/H, 6 D
HD#27	57	F	neg	SI 1 B/C/F
HD#28	64	F	pos	1 A/C-I, 4 C, 6 D, SI 1 B-F
HD#29	58	F	neg	SI 1 B/C/F
HD#30	62	F	neg	SI 1 B/C/F
HD#31	58	F	neg	SI 1 B/C/F
HD#32	74	F	pos	1 A/C-I, 4 C, 6 D, SI 1 B-F
HD#33	73	F	neg	SI 1 B/C/F
HD#34	74	M	neg	SI 1 B/C/F
HD#35	82	M	neg	SI 1 B/C/F
HD#36	82	F	pos	SI 1 B-F SI 2, SI 9

Table S3. Study cohort of patients with liver cirrhosis.
M: male, F: female, AIH: autoimmune hepatitis, ASH: alcohol induced steatohepatis, HBV: hepatitis B virus, HCV: hepatitis C virus, HFE: hemochromatosis, NASH: non-alcoholic steatohepatis, neg: negative, pos: positive, n.d.: not determined.
If not stated otherwise each donor is included in every subsection of the figures.

Patient ID	Age [years]	Sex	Etiology	Child score	GPT [U/l]	GOT [U/l]	HCMV serotatus	Inclusion in figures
cirrhosis#1	53	M	ASH	A	36	54	pos	1, 4 A/C/E, 5, SI 1 B-F, SI 2, SI 9, SI 10
cirrhosis#2	45	M	HBV, HCV	A	31	25	pos	1, 4 A/C/E, 5 A/C-F, SI 1 B-F SI 2, SI 9, SI 10 A-D, G-L
cirrhosis#3	62	M	ASH	C	60	84	pos	1, 4 C/E, 5, SI 1 B-F, SI 9 A/B, SI 10
cirrhosis#4	48	F	HCV	B	46	n.d.	pos	1, 4 C/E, 5, SI 1 B-F SI 2, SI 9, SI 10
cirrhosis#5	49	F	HCV, ASH	A	50	20	pos	1, 4 C/E, 5, SI 1 B-F, SI 2, S 9, SI 10
cirrhosis#6	64	F	HBV	B	28	36	pos	1, 4 C/E, 5, SI 1 B-F, SI 2, SI 9, SI 10
cirrhosis#7	49	F	ASH	A	15	32	pos	1 A/C-I, 4 A/C/E, 5 A/C-F, SI 1 B-F, SI 10 A-D, G-L
cirrhosis#8	55	M	AIH	A	107	65	pos	1, 4 A/C, SI 1 B-F
cirrhosis#9	56	M	HCV	A	25	34	pos	1, 4 A/C/E, SI 1 B-F
cirrhosis#10	61	F	ASH	A	18	28	neg	SI 1 B/C/F
cirrhosis#11	60	F	NASH	B	n.d.	n.d.	pos	1, 4 A/C/E, SI 1 B-F
cirrhosis#12	61	F	ASH	B	20	57	neg	SI 1 B/C/F
cirrhosis#13	73	F	NASH	C	44	56	pos	1, 4 A/C/E, SI 1 B-F
cirrhosis#14	61	F	HCV	A	20	36	pos	1, 4 A/C/E, 5 A/D-F, SI 1 B-F, SI 2, SI 9, SI 10 A/B/G-L
cirrhosis#15	58	M	NASH	B	58	44	pos	1, 4 A/C/E, SI 1 B-F
cirrhosis#16	74	M	NASH	C	n.d.	n.d.	pos	1, 4 A/C/E, 5 D/E, SI 1 B-F, SI 2, SI 9, SI 10 G/H/K/L

cirrhosis#17	68	M	ASH	B	55	48	neg	SI 1 B/C/F
cirrhosis#18	56	M	ASH	B	18	n.d.	pos	1, 4 A/C/E, 5 E/D, SI 1 B-F, SI 10 G/H/K/L
cirrhosis#19	59	M	HCV	A	18	27	neg	SI 1 B/C/F
cirrhosis#20	57	M	NASH	C	26	52	pos	1 A/C-I, SI 1 B-F
cirrhosis#21	76	M	HFE/ drug-toxic	B	1432	667	pos	1 A/C-I, SI 1 B-F
cirrhosis#22	56	M	HBV	C	n.d.	n.d.	pos	1 A/C-I, 4 c, SI 1 B-F
cirrhosis#23	71	F	ASH	B	39	59	pos	1 A/C-I, 4 c, SI 1 B-F
cirrhosis#24	59	M	ASH	B	38	82	neg	SI 1 B/C/F
cirrhosis#25	68	M	secondary biliary cirrhosis	C	n.d.	n.d.	pos	1 A/C-I, 4 C, SI 1 B-F
cirrhosis#26	71	M	NASH	C	88	58	pos	1 A-H, 4 C/E, SI 1 B-F
cirrhosis#27	76	F	NASH	A	21	32	n.d.	SI 1 B/C/F
cirrhosis#28	78	F	HCV	B	n.d.	n.d.	neg	SI 1 B/C/F
cirrhosis#29	76	M	ASH	A	9	19	pos	1 A-H, 4 C/E, SI 1 B-F, SI 2, SI 9
cirrhosis#30	71	F	ASH	A	19	22	pos	1 A-H, 4 C/E, SI 1 B-F
cirrhosis#31	74	M	alpha 1-antitrypsin deficiency	C	n.d.	n.d.	pos	1 A-H, 4 C/E, SI 1 B-F, SI 2 A/B/D/E
cirrhosis#32	68	M	ASH	A	19	27	neg	SI 1 B/C/F
cirrhosis#33	66	M	NASH	A	29	17	neg	SI 1 B/C/F

Table S4. Study cohort of patients with chronic HBV infection (HBV).
M: male, F: female, pos: positive, n.d.: not determined, VL: viral load, Fig.: Figure, SI: Supplementary information

Patient ID	Age	Sex	GPT (U/l)	GOT (U/l)	HCMV serostatus	Geno-type	Therapy	VL (IU/ml)	Inclusion in Figures
HBV#01	55	M	29	23	pos	D	Tenofovir	<10	6 E-G
HBV#02	65	F	34	31	pos	A	naive	2262	6 E/F/G, SI 12 A
HBV#03	36	M	34	28	pos	n.d.	naive	<10	6 E/F/G, SI 12 A
HBV#04	36	M	55	33	pos	D	naive	1149495	6 E-G
HBV#05	25	F	21	20	pos	E	currently no therapy	4002	6 E/F/G, SI 12 A
HBV#06	22	M	27	n.d.	pos	n.d.	naive	534	6 E/F/G, SI 12 A/B
HBV#07	20	F	19	24	pos	A	Tenofovir	13	6 E/F/G, SI 12 A/B
HBV#08	60	M	33	34	pos	n.d.	naive	131	6 F/G, SI 12 A/B
HBV#09	35	F	32	n.d.	pos	A	naive	674	6 F/G, SI 12 A/B
HBV#10	27	F	35	n.d.	pos	n.d.	naive	n.d.	6 F/G, SI 12 A/B

HBV#11	56	M	41	25	pos	n.d.	naive	180	6 F/G, SI 12 A/B
HBV#12	29	F	65	n.d.	pos	n.d.	Tenofovir	1345	6 G, SI 12 A/B
HBV#13	22	F	40	33	pos	n.d.	naive	191	6 F/G, SI 12 A/B
HBV#14	31	M	89	44	pos	D	naive	2822779	6 G
HBV#15	35	M	223	143	pos	E	naive	n.d.	6 F/G
HBV#16	39	F	12	18	pos	n.d.	Tenofovir	<10	6 G, SI 12 A/B
HBV#17	43	M	17	19	pos	n.d.	naive	n.d.	6 G
HBV#18	50	F	22	31	pos	n.d.	naive	2548	6 G
HBV#19	40	M	47	37	pos	Ba	Entecavir	55	6 G, SI 12 B
HBV#20	45	F	17	20	pos	B	naive	13467	6 G, SI 12 A/B
HBV#21	43	F	9	n.d.	pos	n.d.	naive	10	6 E-G, SI 12 A
HBV#22	42	M	36	21	pos	D	naive	19227	6 E-G
HBV#23	53	M	109	86	pos	n.d.	Adefovir	<10	6 E-G
HBV#24	26	M	56	n.d.	pos	E	Tenofovir	55	6 E-G
HBV#25	43	M	34	23	pos	n.d.	Tenofovir	<10	6 E-G
HBV#26	59	F	20	28	pos	n.d.	Telbivudin	<10	6 E-G
HBV#27	62	M	64	41	pos	n.d.	Tenofovir	n.d.	6 E-G
HBV#28	47	M	31	30	pos	n.d.	Tenofovir	<10	6 E-G

Table S5. Study cohort of liver and tumor samples from the hepatologic and gastroenterologic biobank of the University hospital Freiburg.

Data is depicted in figures 2, 4 SI figures 3-5.

For detailed information about the patients see SI table 1, HCC#53-60.

HCC: Hepatocellular carcinoma, SI: Supplementary information.

Patient ID	Samples available	Inclusion in Figures
HCC#53	PBMCs, non-tumoral liver, HCC tumor tissue	SI 3, 2 A-E, 4 B/D/F
HCC#54	PBMCs, non-tumoral liver, HCC tumor tissue	SI 3, 2 A-E, 4 B/D/F
HCC#55	PBMCs, HCC tumor tissue	SI 3, 2 A-E, 4 B/D/F
HCC#56	PBMCs, non-tumoral liver, HCC tumor tissue	2 A-E/G, 4 B/D/F, SI 3, SI 4 B/E/H, SI 5 B/E/H/K,
HCC#57	PBMCs, non-tumoral liver, HCC tumor tissue	2, 4 B/D/F, SI 3 A-D SI 4, SI 5
HCC#58	PBMCs, non-tumoral liver, HCC tumor tissue	2 B/F/G/H, 4 B, SI 3 A/B, SI 4, SI 5,
HCC#59	PBMCs, non-tumoral liver, HCC tumor tissue	2 B/F/G/H, 4 B, SI 3 A/B, SI 4, SI 5
HCC#60	PBMCs, non-tumoral liver, HCC tumor tissue	SI 3 A/B, SI 4, SI 5 A-I

Table S6. Study cohort of liver samples obtained from the Karolinska Institute, Sweden.

All patients were subjected to liver resection for metastasis of colorectal cancer. Only non-tumoral liver was obtained and subsequently analyzed.

Data is depicted in SI figures 3, 5 and 9.

M: male, F: female, pos: positive, SI: Supplementary information

If not stated otherwise each donor is included in every subsection of the figures.

Patient ID	Age	Sex	HCMV serostatus	Tissue samples	Inclusion in figures
Non-tumoral Liver#1	68	M	pos	Non-tumoral liver	SI 3, SI 5
Non-tumoral Liver#2	74	F	pos	Non-tumoral liver	SI 3, SI 5
Non-tumoral Liver#3	71	M	pos	Non-tumoral liver	SI 3, SI 5, SI 11 B
Non-tumoral Liver#4	73	M	pos	Non-tumoral liver	SI 3 A-D, SI 5
Non-tumoral Liver#5	78	F	pos	Non-tumoral liver	SI 3 A-C, SI 5 A/C/D/F/G/I/J/L, SI 11 A
